# The effect of vitamin C supplementation on lipid profile of type 2 diabetic patients: a systematic review and meta-analysis of clinical trials

**DOI:** 10.1186/s13098-021-00640-9

**Published:** 2021-03-02

**Authors:** Amare Abera Tareke, Addis Alem Hadgu

**Affiliations:** 1 Physiology Unit, Department of Biomedical Sciences, College of Medicine and Health Sciences, Wollo University, Dessie, Ethiopia; 2 Biochemistry Unit, Department of Biomedical Sciences, College of Medicine and Health Sciences, Wollo University, Dessie, Ethiopia

**Keywords:** Diabetes mellitus, Dyslipidemia, Lipid profiles, Meta-analysis, Vitamin C

## Abstract

**Background and aims:**

We conducted a systematic review and meta-analysis of clinical trials evaluating the role of vitamin C supplementation on lipid profiles among diabetic patients to summarize the available findings.

**Methods:**

A comprehensive search of PubMed, ScienceDirect, Google Scholar, and Cochrane Library databases was performed. Clinical trials conducted on adult type 2 diabetic patients evaluating the effect of vitamin C supplementation and reported lipid profiles (cholesterol (TC), triglycerides (TG), low density lipoprotein (LDL), high density lipoprotein (HDL)) were included. Weighted mean difference (WMD) was calculated.

**Results:**

Vitamin C supplementation had no significant effect on TC (WMD = − 4.36 mg/dl (95% CI − 10.24, 1.52) p-value = 0.146), LDL level (WMD = 2.73 mg/dl (95% CI − 1.72, 7.17) p-value = 0.229), and HDL level (WMD = 0.91 mg/dl (CI − 0.45, 2.27) p-value = 0.191). However, it reduced TG and secondary outcomes (FBS and HgA1C): TG (WMD = − 11.15 mg/dl (95% CI − 21.58, − 0.71) p-value = 0.036), FBS (WMD = − 16.94 mg/dl CI − 21.84, − 12.04, p-value = 0.000), and HgA1C (WMD = − 1.01% CI − 1.18, − 0.83, p-value = 0.001. Subgroup analysis also depicted younger patients, longer duration of treatment and higher dose were important factors. In addition, meta-regression analysis indicated the significant role of patient age, duration of treatment, supplementation dose, BMI and other baseline variables.

**Conclusion:**

There is no adequate evidence to support vitamin C supplementation for dyslipidemias in diabetic patients. Specific group of patients might have benefited including younger diabetic patients. Future researches should give emphasis on the duration of treatment, the dose of vitamin C and baseline values.

**Supplementary Information:**

The online version contains supplementary material available at 10.1186/s13098-021-00640-9.

## Background

Association of insulin resistance with elevated total triglycerides (TG) and low-level high-density lipoprotein cholesterol (HDL-C) is well established in many former studies [1–3]. Lipoprotein abnormalities such as small dense low-density lipoprotein (LDL) and excess TG rich remnants were attributed to insulin resistance [4]. In light of the insulin resistance in Type 2 Diabetes Mellitus (T2DM), dyslipidemia is a common problem in these patients. Worsening dyslipidemia and inflammation overtime among T2DM patients rise concern regarding the premature development of atherosclerosis [5].

Insulin resistance with attendant increase free fatty acid flux into the liver pays a central role in triad of dyslipidemias in the form of high TG and high LDL-C and low HDL-C [6]. Other pathogenic mechanism of diabetic dyslipidemia relates to the increased production of inflammatory cytokines such as tumor necrosis factor-a. These cytokines increase insulin resistance and down regulate HDL production while increasing the activity of key enzymes promoting hypertriglyceridemia [7, 8].

The prevalence of dyslipidemia in diabetic patients ranges up to 90%, especially in patients with poor glycemic control [9]. Lipids are classically associated with cardio vascular disease (CVD) and they are involved in diabetic microvascular complications. Elevated total cholesterol (TC) and TG levels have deleterious effects on kidney resulted in nephropathy [10, 11], whereas, increase in HDL level is suggested to be protective of renal injury [12]. Development of retinal hard exudate with diabetic macular edema has been noticed associated with elevated serum lipids [13]. Diabetic neuropathy with possible vascular etiology was pointed out as result of dyslipidemia [14]. The proportion of diabetic patients receiving lipid lowering agents is increasing [15] indicating the urge to decrease lipids in these patients.

Ascorbic acid is a water soluble vitamin with antioxidant activity, which, can also regenerate other antioxidants like vitamin E [16]. However, some in vivo studies implicated potential pro-oxidative effects of this vitamin [17, 18]. Patients with diabetes have lower circulating levels of vitamin C and higher level of lipid peroxides, reports also suggest improvement in insulin action, glycemic control and endothelial function [19]. Contrary to these findings, high dose vitamin C may increase the rate of cardiovascular complications, as observed in postmenopausal women [20]. Such findings are inconclusive to synthesize scientific evidence.

Besides clinical trials conducted from 1980′s till now, we failed to retrieve a systematic review and meta-analysis study summarizing the effects of vitamin C supplementation on lipid profile of T2DM patients. Thus, we aimed to conduct a comprehensive systematic review and meta-analysis of clinical trials to summarize the available findings.

## Methods

### Literature searches

A comprehensive search of PubMed, ScienceDirect, Google Scholar, and Cochrane Library databases was performed. Reports were compiled according to the prefered reporting items for systematic reviews and meta-analysis (PRISMA) guideline [21]. Unpublished articles were searched from clinical trials registration platforms. Preprint articles were also retrieved from websites. Manual search was conducted by screening the reference lists of inclusive studies. The search strategy involves patients with diabetes mellitus and intervention relevant terms including vitamin C or ascorbic acid with both Medical Subject Headings (MeSH) and free text. The following search terms were used with Boolean search operator. Diabetes, “diabetes mellitus”, “type 2 DM, DM, “non-insulin dependent diabetes mellitus”, “late onset diabetes mellitus” vitamins, “vitamin C”, “ascorbic acid”, “antioxidant vitamins”, “lipid profile”, “lipid indices”, cholesterol, “total cholesterol”, triglycerides, “high density lipoproteins” HDL, “low density lipoproteins” LDL. All relevant articles published or unpublished available until the commencement of the study were included.

### Study selection and outcomes

#### Inclusion criteria

The studies were considered for inclusion if they are clinical trials, performed among adult patients with T2DM; evaluating the effect of vitamin C supplementation; reporting mean and standard deviation, median with maximum and minimum or median with 1^st^ and 3^rd^ quartiles of lipid profiles (TC, TG, LDL, HDL) of intervention and control groups. The time of intervention should be at least two weeks. Due to feasibility, articles published in English language/have English version was included. All above mentioned researches conducted on adults and before May 1, 2020 was included.

#### Exclusion criteria

The control group should be placebo or no treatment at all, studies which have non-placebo control group (active substance) were excluded. Cross over trials were excluded from this meta-analysis. Studies lacking control group or with non-diabetic control were excluded. This meta-analysis excluded in vitro and animal studies, review articles, studies with cohort, case control or cross-sectional design, trials with combination therapy (combine vitamin C with other nutrients). Incomplete articles, conference procedings, and duplicates was excluded. The two authors independently screened the titles, abstracts and full-text of retrieved articles to identify their eligibility and disagreements were resolved by discussion and consensus among the two authors.

#### Outcome variables

The primary outcome of the current study was to assess the effect of vitamin C supplementation on lipid profile [measured in terms of TC, TG, HDL and LDL] among type 2 DM patients. As secondary outcomes we also evaluated the effect of vitamin C on FBS and HgA1C among those patients.

### Data extraction and quality assessment

Data extraction were conducted by the two authors independently using a predefined data extraction format. Relevant variables extracted were study characteristics, population characteristics, intervention characteristics, and outcomes (TC, TG, LDL, HDL, FBS and HgA1C). Disagreement on data extraction between the two investigators was solved by discussion and consensus. The risk of bias of inclusive studies were assessed in accordance with the Cochrane Collaboration Risk of Bias Tool [22]. The risk of bias of individual study was rated as low, moderate, or high.

### Statistical analysis

Since the outcome variables were continuous variables, weighted mean difference (WMD) with 95% confidence interval (CI) was calculated using random effects, inverse variance model. I squared statistic (I^2^) was used to measure heterogeneity, with a I^2^ > 50% representing notable heterogeneity [23]. Subgroup analysis was performed based on the dose of vitamin C, duration of supplementation and participants mean age.

For statistical feasibility (considering the number of studies in each subgroup) the dose of vitamin C was categorized in to two; (1) less than 1000 mg/day and (2) greater than or equal to 1000 mg/day. The duration of supplementation was dichotomized in to less than 12 weeks and greater than or equal to 12 weeks. Whereas, the mean age of participants was categorized after calculating grand mean, which was 52.87 years. Studies reported median with maximum and minimum or median with 1st and 3rd quartiles of lipid profiles (TC, TG, LDL, HDL) and FBS or HgA1C of intervention and control groups converted into means and standard deviation according to Wan et al. [24]. For studies which have more than one experimental arms, the sample size of the control group is adjusted by dividing the control group sample by the number of experimental arms. 

To identify possible covariates, meta-regression analysis was performed for each primary and secondary outcome of the study. Possible covariates were baseline measurements (Tc, TG, HDL, LDL, FBS, HgA1C, body mass index (BMI)), dose, duration and age. For detection of robustness of the results sensitivity analysis was conducted by sequential elimination of each study from the pool. The sensitivity analysis was conducted for each primary and secondary outcome. Potential publication bias was assessed by visual inspection of funnel plot, and considering the number of studies for each outcome we claimed egger’s regression test for bias detection. All statistical analysis was performed using STATA software Version 16. (Statacorp, Texas, USA) with p-value P < 0.05 indicating a statistically significant difference of two-sided test.

## Results

### Search result

**Fig. 1:**
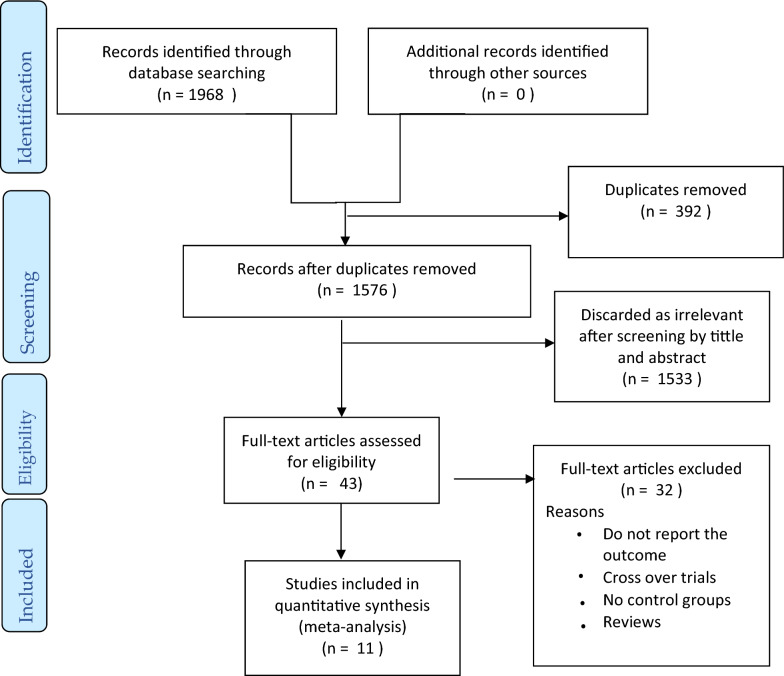
PRISMA flow diagram of study selection to determine the effect of vitamin C on lipid profile among diabetic patients

A total of 1968 articles were retrieved after searching the four databases [PubMed (562), Google Scholar (1190), Cochrane Library (71), and ScienceDirect (145)]. Three hundred ninety-two duplicates were removed, after screening title and abstract another 1533 articles were excluded. Forty-three articles were selected for full text screening. With various exclusions, finally 11 articles selected for this meta-analysis. Exclusions were wrong control group; no control group (2 studies), non-diabetic control (1), healthy control (1). Supplementation given in combination with; vitamin E (5), zinc (1), gemfibrozil (1), green tea and pomegranate extract (1). Others were cross over trials (6), fail to report outcome (6), animal study (1), conducted on other patients [hypertension (1), metabolic syndrome (1)], incomplete articles; abstract (1), reported only median and IQR (1), no standard deviation (1), and review (2). The summary of selection process is given in Fig. 1.

### Characteristics of included studies

Eleven studies [25–35], conducted between 2003 and 2020 included a total of 606 T2DM patients and their mean age of participants in the studies ranged 37.8–60 years. The dose of vitamin C supplementation ranged 200 mg/day to 2000 mg/day. The minimum duration of supplementation was 4 weeks and the maximum were 52 weeks. All 11 studies reported TC, LDL, HDL and FBS, and 10 studies reported TG and HgA1C. Characteristics of included studies summarized in Table 1.

**Table 1:** Characteristic of included studies

Authors	Year	Sample size	Age	Dose	Duration	Baseline values						
						FBS	TC	TG	LDL	HDL	HgA1C	BMI
Bhatt et al.[25]	2013	59	57.5	500	12	NR	196.83	160.31	96.67	51.98	NR	25.8
El Al et al.[26]	2018	20	51.02	1000	12	154.88	209.75	131.5285	122.45	22.91	8.065	27.87
Evans et al.[27]	2003	20	53.2	1000	6	157.5	210.75	103.24	129.54	27.65	8.45	28.9
Ghaffari et al.[28]	2015	30	51.9	800	8	176.93	227.35	NR	159.93	43.48	NR	NR
Gilliani et al.[29]	2017	210^a^	37.8	500	52	142.06	233.59	84.77	120.08	37.9	9.15	23.90
Hemed et al.[30]	2016	39	52	1000	12	143.81	239.38	123.94	157.12	34.35	7.29	NR
Mahmoudabadi et al.[31]	2011	23	51.45	200	8	154.02	163.6	148.92	100.7	40.12	7.91	29.1
Rafighi et al.[32]	2011	58	53.82	266.7	12	151.75	191.21	122.24	129.62	35.11	8.39	29.86
Ragheb et al. [33]	2020	27	55.16	500	8	143.89	230.72	105.22	138.41	62.63	8.64	33.57
Sanguanwong et al.[34]	2016	100	57.75	1000	8	148.63	172.05	75.13	89.73	41.34	7.72	25.74
Tousoulis et al.[35]	2007	20^a^	60	2000	4	NR	182.13	81.33	103.24	32.09	6.48	28.5

*BMI* body mass index, *FBS* fasting blood sugar, *HDL*: high density lipoprotein, *HgA1C* hemoglobin A1C, *LDL* low density lipoprotein, *TC* total cholesterol, *TG* total triglycerides, *NR* not reported

^a^The sample size of the studies were corrected based on the number of experimental arms as indicated in methodology

### Meta-analysis

#### Vitamin C and TC

**Fig. 2:**
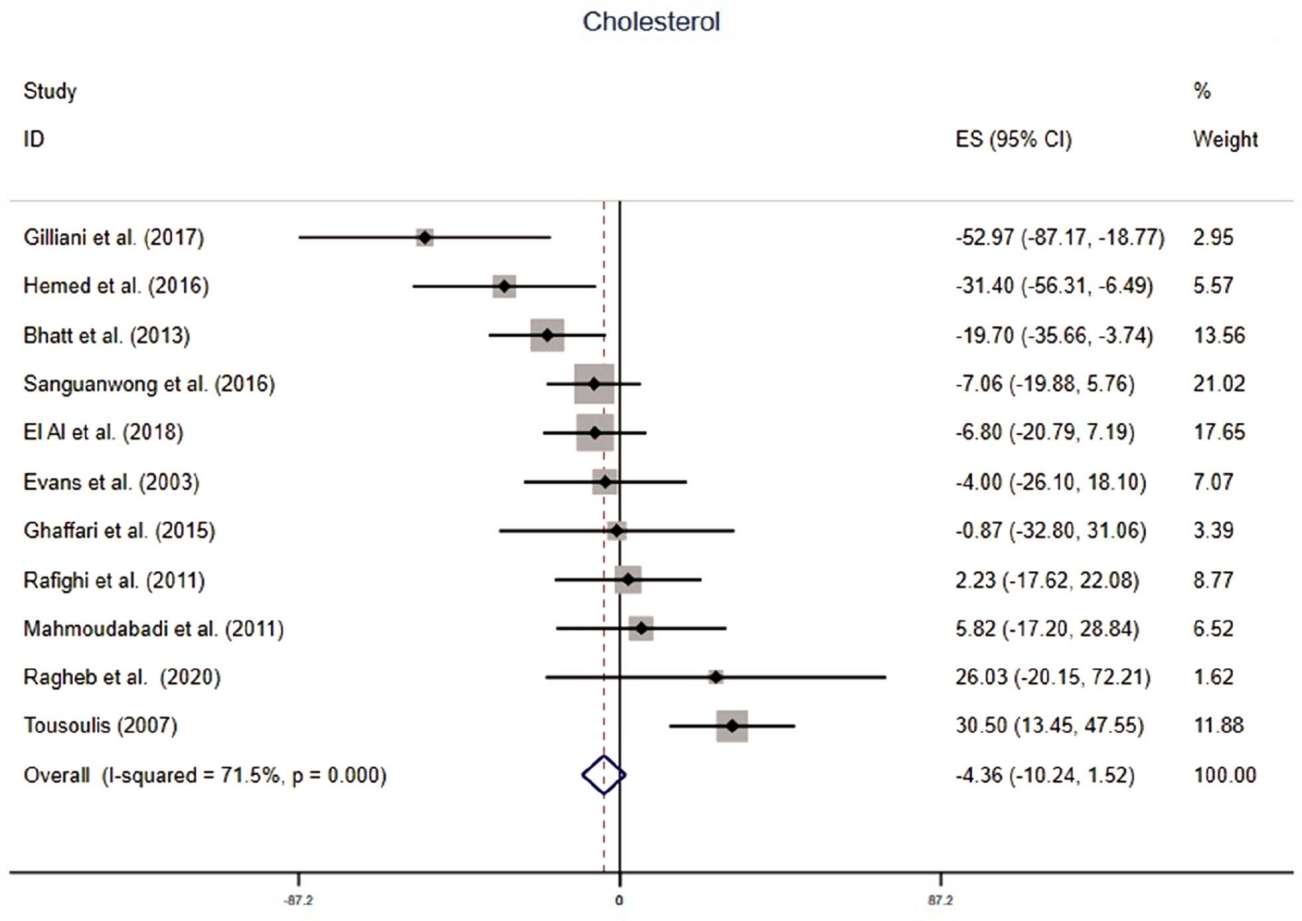
The effect of vitamin C supplementation of serum cholesterol among type 2 diabetic patients. There was no significant reduction in total cholesterol due to vitamin C. With moderate heterogeneity, the weighted mean difference was − 4.36 mg/dl CI − 10.24–1.52 and p-value = 0.146

**Fig. 3:**
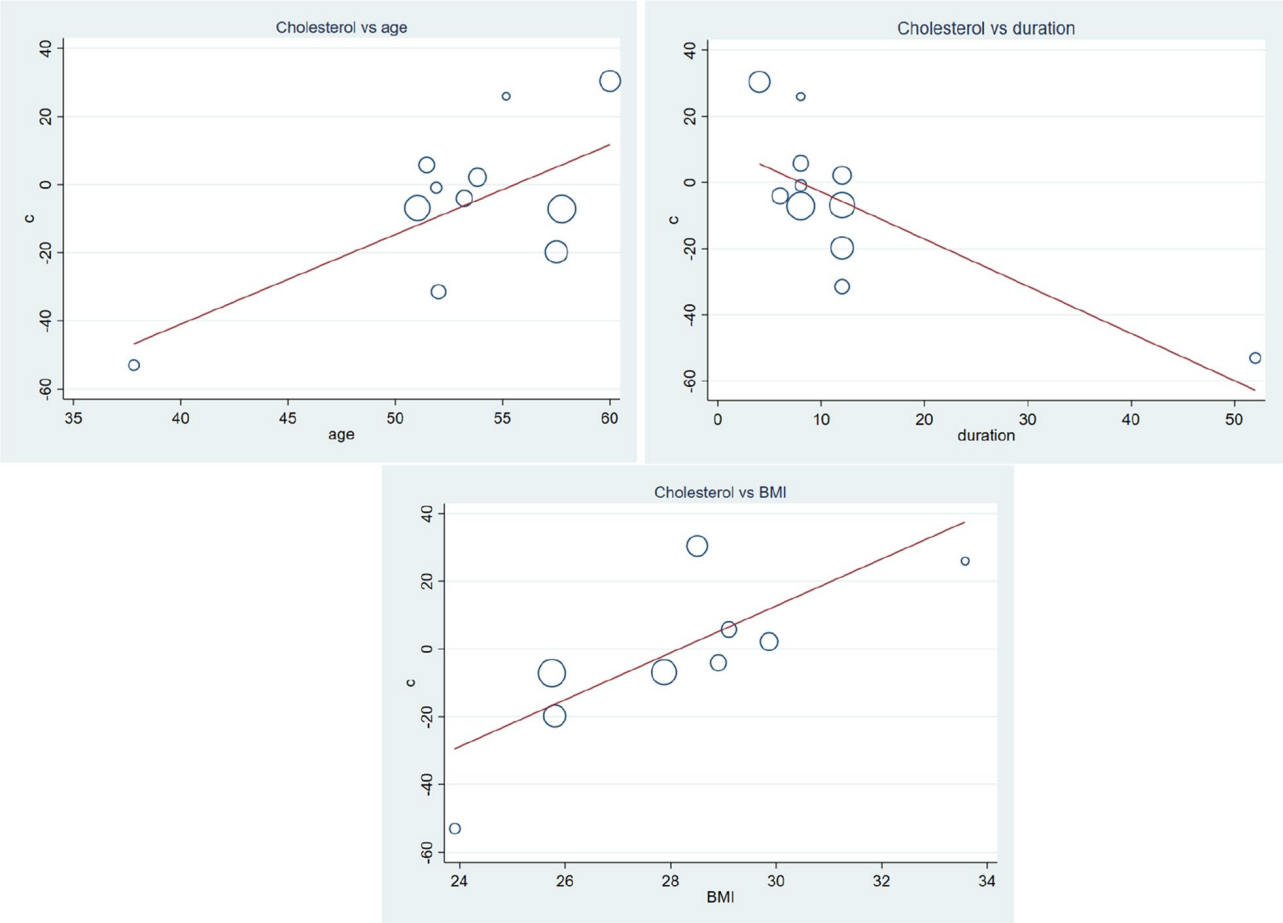
Meta-regression analysis of the effect of vitamin C supplementation on serum cholesterol of type 2 diabetic patients; the influence of covariates patients mean age, duration of supplementation and baseline BMI. As indicated, WMD increases with increment in age, BMI and decrement in duration. The higher reduction was observed in younger type 2 DM patients (β = 2.63, CI 0.22–5.04, p-value = 0.036), longer duration of treatment (β = -1.42, CI − 2.48, − 0.36, p-value = 0.014), and lower BMI (β = 6.92, CI 1.02–12.82, p-value = 0.028)

Supplementation of vitamin C fail to decrease serum TC, WMD = − 4.36 mg/dl (95% CI − 10.24, 1.52) p-value = 0.146 with moderate heterogeneity, I^2^ = 71.5% (Fig. 2). Subgroup analysis indicated vitamin C supplementation may have beneficial effect in younger T2DM patients WMD = − 11.54 (95% CI − 21.32, − 1.75) p-value = 0.021 (Table 2). In longer treatment periods (> 12 weeks), vitamin C significantly decreased serum TC WMD = − 14.41 mg/dl (95% CI − 22.65, − 5.97) p-value = 0.001. Furthermore, meta-regression analysis showed mean of age, duration of treatment and baseline BMI have influence of WMD. Whereas, baseline FBS, TC, TG, LDL, HDL and HgA1C had no influence on mean difference of TC (Table 3). Vitamin C supplementation have greater benefit on younger patients (β = 2.63, CI 0.22–5.04, p-value = 0.036), longer duration of treatment (β = − 1.42, CI − 2.48, − 0.36, p-value = 0.014), and lower BMI (β = 6.92, CI 1.02–12.82, p-value = 0.028) (Fig. 3). Sensitivity analysis depicted omitting one study, gives a significant WMD = − 9.06 (95% CI − 15.32, − 2.79) (Additional file 1: Fig. S1 and Table S1).

**Table 2:** Subgroup analysis of outcome variables based on mean age, treatment duration and daily vitamin C dose

Outcome		Subgroup	Studies	Q statistic	I^2^(p-value)	Z	ES(95% CI)	Inter-group I^2^-P
TC	Total		11	35.07	71.5(0.000)	1.45(0.146)	− 4.36(− 10.24, 1.52)	
	Age	< 52.8 years	5	11.13	61.1(0.025)	2.31(0.021)	− 11.54(− 21.32, − 1.75)	0.072
		> 52.8 years	6	20.7	75.8(0.001)	0.08(0.935)	− 0.31(− 7.66, 7.04)	
	Dose	< 1000 mg	6	13.36	62.6(0.020)	1.8(0.072)	− 8.88(− 18.57, 0.81)	0.250
		≥ 1000 mg	5	20.39	80.4(0.000)	0.46(0.647)	− 1.73(− 9.12, 5.67)	
	Duration	≤ 12 week	6	13.56	63.1(0.019)	1.22(0.222)	5.1(− 3.09, 13.29)	0.001
		> 12 week	5	10.93	63.4(0.027)	3.35(0.001)	− 14.41(− 22.65, − 5.97)	
TG	Total		10	63.02	85.7(0.000)	2.09(0.036)	− 11.15(− 21.58, − 0.71)	
	Duration	≤ 12 week	5	37.73	89.4(0.000)	1.73(0.084)	14(− 1.89, 29.90)	0.000
		> 12 week	5	8.39	52.3(0.078)	4.28(0.000)	− 30.19(− 44.02, − 16.36)	
	Dose	< 1000 mg	5	54.82	92.7(0.000)	0.66(0.509)	− 5.35(− 21.24, 10.53)	0.343
		≥ 1000 mg	5	7.30	45.2(0.121)	2.20(0.028)	− 15.55(− 29.38, − 1.71)	
	Age	< 52.8	4	7.31	59(0.063)	4.15(0.000)	− 39.66(− 58.38, − 20.95)	0.000
		> 52.8	6	42.77	88.3(0.000)	0.27(0.789)	1.71(− 10.86, 14.28)	
LDL	Total		11	118.65	91.6(0.000)	1.2(0.229)	2.73(− 1.72, 7.17)	
	Dose	< 1000 mg	6	15.90	68.6(0.007)	4.58(0.000)	− 17.77(− 25.37, − 10.17)	0.000
		≥ 1000 mg	5	60.34	93.4(0.000)	4.78(0.000)	13.35(7.88, 18.82)	
	Duration	≤ 12 week	6	40.03	87.5(0.000)	6.04(0.000)	18.2(12.3, 24.11)	0.000
		> 12 week	5	17.82	77.5(0.001)	5.07(0.000)	− 17.45(− 24.19, − 10.71)	
	Age	< 52.8	5	22.77	82.4(0.000)	2.96(0.003)	− 12.98(− 21.57, − 4.40)	0.000
		> 52.8	6	78.33	93.6(0.000)	3.2(0.001)	8.46(3.27, 13.65)	
HDL	Total		11	35.11	71.5(0.000)	1.31(0.191)	0.91(− 0.45, 2.27)	
	Dose	< 1000 mg	6	18.11	76.4(0.002)	1.07(0.284)	1.07(− 0.89, 3.02)	0.822
		≥ 1000 mg	5	16.95	72.4(0.003)	0.78(0.434)	0.76(− 1.14, 2.65)	
	Duration	≤ 12 week	6	8.32	39.9(0.139)	1.54(0.122)	− 2.01(− 4.57, 0.54)	0.008
		> 12 week	5	19.79	79.8(0.001)	2.51(0.012)	2.06(0.45, 3.67)	
	Age	< 52.8	5	16.19	75.3(0.003)	2.94(0.003)	3.53(1.18, 5.88)	0.007
		> 52.8	6	11.72	57.3(0.039)	0.49(0.627)	− 0.41(− 2.08, 1.25)	
FBS	Total		11	50.88	80.3(0.000)	6.77(0.000)	− 16.94(− 21.84, − 12.04)	
	Dose	< 1000 mg	6	20.72	75.9(0.001)	3.46(0.001)	− 17.49(− 27.39, − 7.59)	0.900
		≥ 1000 mg	5	30.4	86.7(0.000)	5.82(0.000)	− 16.76(− 22.40, − 11.11)	
	Duration	≤ 12 week	6	5.03	0.61(0.412)	1.12(0.246)	− 3.96(− 10.90, 2.98)	0.000
		> 12 week	5	19.04	79.0(0.001)	8.45(0.000)	− 29.86(− 36.79, − 22.93)	
	Age	< 52.8	5	20.71	80.7(0.000)	8.34(0.000)	− 29.53(− 36.47, − 22.59)	0.000
		> 52.8	6	4.97	0.0(0.419)	1.25(0.211)	− 4.41(− 11.34, 2.51)	
HgA1C	Total		10	85.8	89.5(0.000)	11.2(0.001)	− 1.01(− 1.18, − 0.83)	
	Dose	< 1000 mg	5	63.83	93.7(0.000)	8.6(0.000)	− 1.62(− 1.99, − 1.25)	0.000
		≥ 1000 mg	5	8.32	51.9(0.081)	8.15(0.000)	− 0.83(− 1.03, − 0.63)	
	Duration	≤ 12 week	5	12.17	67.1(0.016)	7.38(0.000)	− 0.8(− 1.02, − 0.59)	0.001
		> 12 week	5	62.87	93.6(0.000)	9.11(0.000)	− 1.43(− 1.74, − 1.12)	
	Age	< 52.8	4	59.7	95(0.000)	9.26(0.000)	− 1.47(− 1.78, − 1.16)	
		> 52.8	6	13.65	63.4(0.018)	7.31(0.000)	− 0.79(− 1.0, − 0.58)	

BMI: body mass index, FBS: fasting blood sugar, HDL: high density lipoprotein, HgA1C: hemoglobin A1C, LDL: low density lipoprotein, TC: total cholesterol, TG: total triglycerides

**Table 3:** Meta-regression analysis result of outcomes among baseline factors, and patients and treatment characteristics

	Covariate	Coefficient (β)	95% CI	P-value
TC	Age	2.63	0.22, 5.04	0.036
	Dose	0.01	− 0.01, 0.04	0.223
	Duration	− 1.42	− 2.48, − 0.36	0.014
	FBS	0.80	− 0.77, 2.39	0.268
	TC	− 0.42	− 0.98, 0.14	0.126
	TG	− 0.11	− 0.73, 0.50	0.675
	LDL	− 0.15	− 0.85, 0.54	0.629
	HDL	0.05	− 1.55, 1.67	0.938
	HgA1C	− 14.96	− 38.56, 8.63	0.177
	BMI	6.92	1.02, 12.82	0.028
TG	Age	4.22	− 3.17, 11.62	0.224
	Dose	− 0.01	− 0.11, 0.08	0.772
	Duration	− 2.10	− 5.20, 1.00	0.158
	FBS	− 1.00	− 12.72, 10.72	0.841
	TC	0.14	− 1.77, 2.06	0.871
	TG	0.05	− 1.67, 1.78	0.946
	LDL	0.48	− 1.84, 2.80	0.647
	HDL	3.36	0.09, 6.63	0.045
	HgA1C	3.72	− 73.66, 81.12	0.912
	BMI	18.35	6.82, 29.88	0.007
LDL	Age	2.85	0.29, 4.82	0.031
	Dose	0.02	0.00, 0.05	0.039
	Duration	− 1.16	− 2.17, − 0.16	0.027
	FBS	1.12	− 0.38, 2.64	0.122
	TC	− 0.53	− 1.13, 0.05	0.071
	TG	− 0.17	− 0.84, 0.49	0.570
	LDL	− 0.31	− 1.06, 0.43	0.366
	HDL	− 0.10	− 1.84, 1.63	0.893
	HgA1C	− 20.77	− 41.32, − 0.22	0.048
	BMI	4.88	− 2.45, 12.21	0.159
HDL	Age	− 1.03	− 1.63, − 0.42	0.004
	Dose	− 0.004	− 0.01, 0.004	0.257
	Duration	0.59	0.29, 0.89	0.001
	FBS	− 0.25	− 0.78, 0.26	0.281
	TC	0.15	− 0.005, 0.31	0.057
	TG	− 0.007	− 0.22, 0.20	0.937
	LDL	0.05	− 0.16, 0.27	0.566
	HDL	− 0.01	− 0.56, 0.54	0.954
	HgA1C	5.70	− 1.77, 13.17	0.114
	BMI	− 2.27	− 4.61, 0.06	0.055
FBS	Age	2.17	0.47, 3.87	0.018
	Dose	0.002	− 0.02, 0.02	0.848
	Duration	− 0.97	− 1.73, − 0.21	0.018
	FBS	0.31	− 1.77, 2.41	0.729
	TC	− 0.20	− 0.74, 0.33	0.416
	TG	0.10	− 0.46, 0.67	0.686
	LDL	0.03	− 0.62, 0.69	0.916
	HDL	0.92	− 0.48, 2.34	0.173
	HgA1C	− 9.04	− 30.38, 12.29	0.349
	BMI	6.29	1.23, 12.61	0.024
HgA1C	Age	0.10	0.01, 0.19	0.032
	Dose	− 0.0001	− 0.001, 0.001	0.964
	Duration	− 0.05	− 0.08, − 0.02	0.003
	FBS	0.13	0.005, 0.262	0.044
	TC	− 0.01	− 0.04, 0.01	0.328
	LDL	− 0.002	− 0.04, 0.04	0.879
	HDL	0.017	− 0.05, 0.09	0.617
	HgA1C	− 0.38	− 1.48, 0.71	0.438
	BMI	0.27	0.00, 0.54	0.049

*BMI* body mass index, *CI* confidence interval, *FBS* fasting blood sugar, *HDL* high density lipoprotein, *HgA1C* hemoglobin A1C, *LDL* low density lipoprotein, *TC* total cholesterol, *TG* total triglycerides. The covariates are baseline factors

#### Vitamin C and TG

**Fig. 4:**
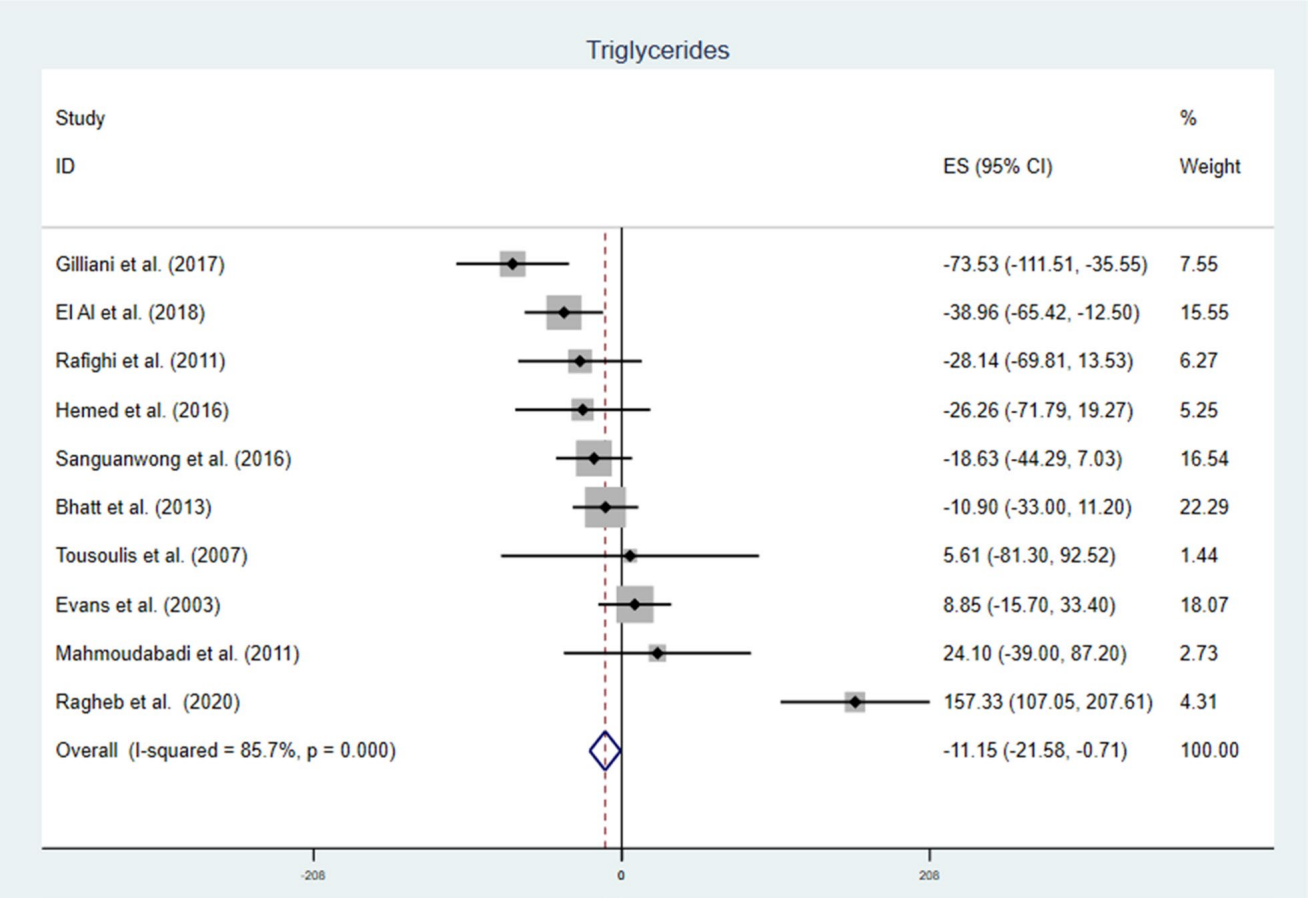
The effect of vitamin C supplementation of serum triglycerides among type 2 diabetic patients. There was a significant reduction in triglyceride level due to vitamin C. With significant heterogeneity, the weighted mean difference was -11.15 mg/dl CI − 21.58, − 0.71 and p-value = 0.036

**Fig. 5:**
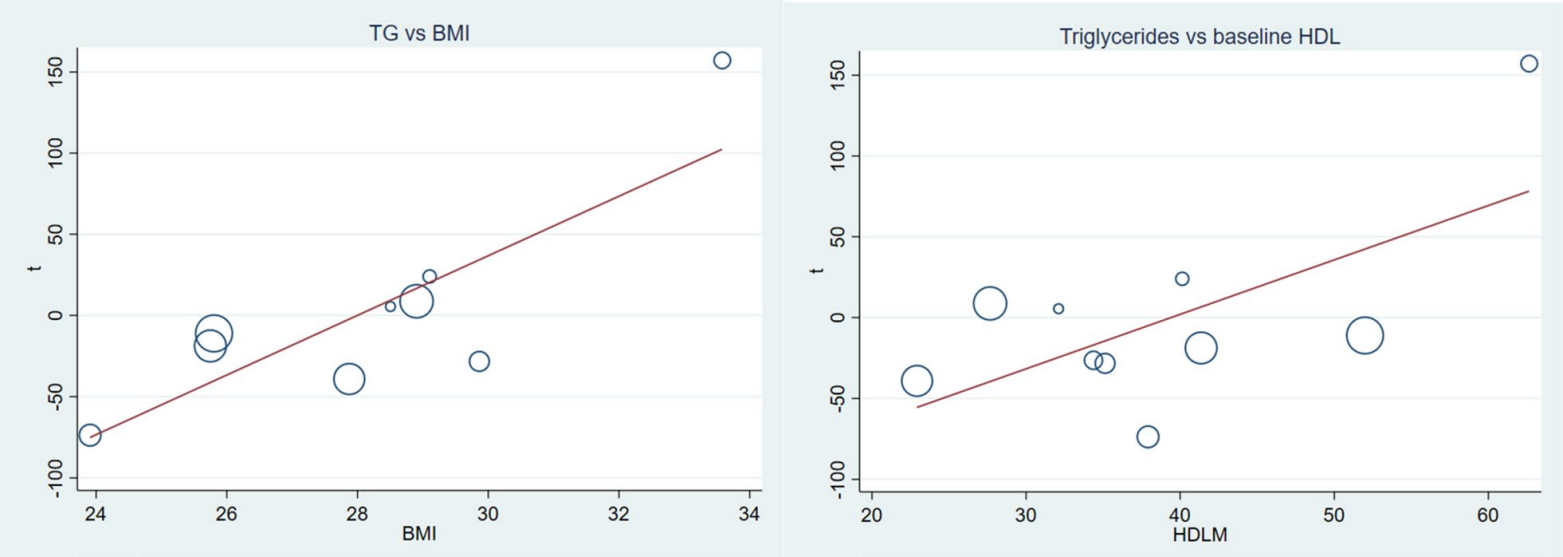
Covariate affecting WMD of triglycerides; baseline BMI and baseline HDL level. HDL (β = 3.36 CI 0.09, 6.63: p-value = 0.045) and BMI (β = 18.35 CI 6.83, 29.88 p-value = 0.007). the reduction was pronounced in lower baseline HDL and BMI

Vitamin C supplementation to T2DM patients significantly reduce serum TG with notable heterogeneity, WMD = − 11.15 mg/dl (95% CI − 21.58, − 0.71) p-value = 0.036 and I^2^ = 85.7 (Fig. 4). Subgroup analysis also pointed out that the reduction was higher with longer duration, higher daily dose and younger patients. Duration > 12 weeks WMD = − 30.19 mg/dl (CI − 44.02, − 16.36), p-value = 0.000, vitamin C daily dose ≥ 1000 mg WMD = − 15.55 mg/dl (CI − 29.38, − 1.71) p-value = 0.028, and mean age < 52.8 WMD = − 39.66 mg/dl (CI − 58.38, − 20.95) p-value = 0.000 (Table 2). Meta-regression analysis (Table 3) showed baseline FBS, TC, TG, LDL, HgA1C, age, dose and duration of supplementation were not affecting TG. Whereas, HDL (β = 3.36 CI 0.09, 6.63: p-value = 0.045) and BMI (β = 18.35 CI 6.83, 29.88 p-value = 0.007) were covariates affecting the effect size (Fig. 5). Sensitivity analysis for TG indicated no single study had significant influence on the outcome (Additional file 1: Fig. S2 and Table S2).

#### Vitamin C and LDL

**Fig. 6:**
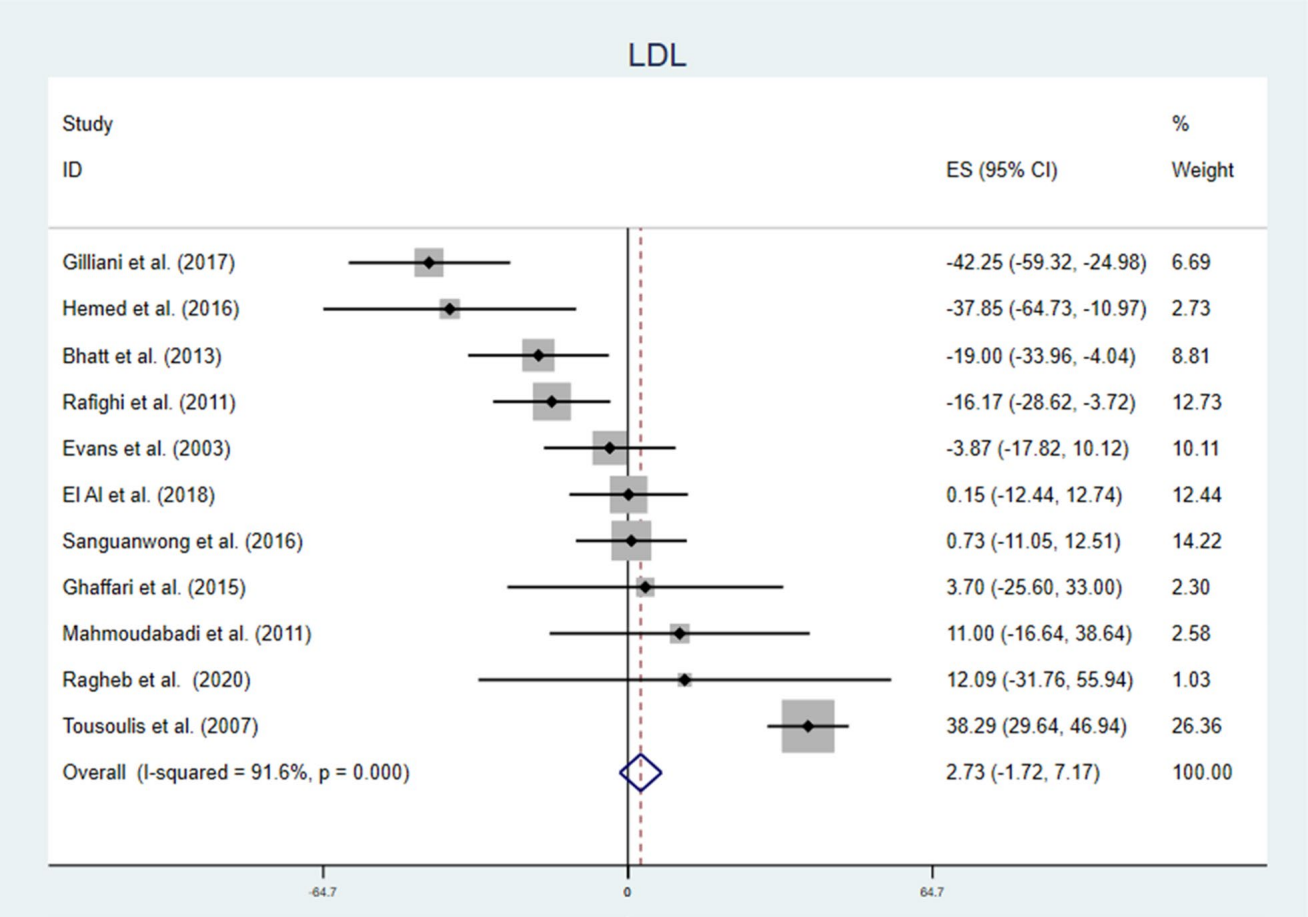
Forest plot showing the effect of vitamin C supplementation on serum LDL among diabetic patients. There was no significant change in serum LDL, there was also significant heterogeneity

**Fig. 7:**
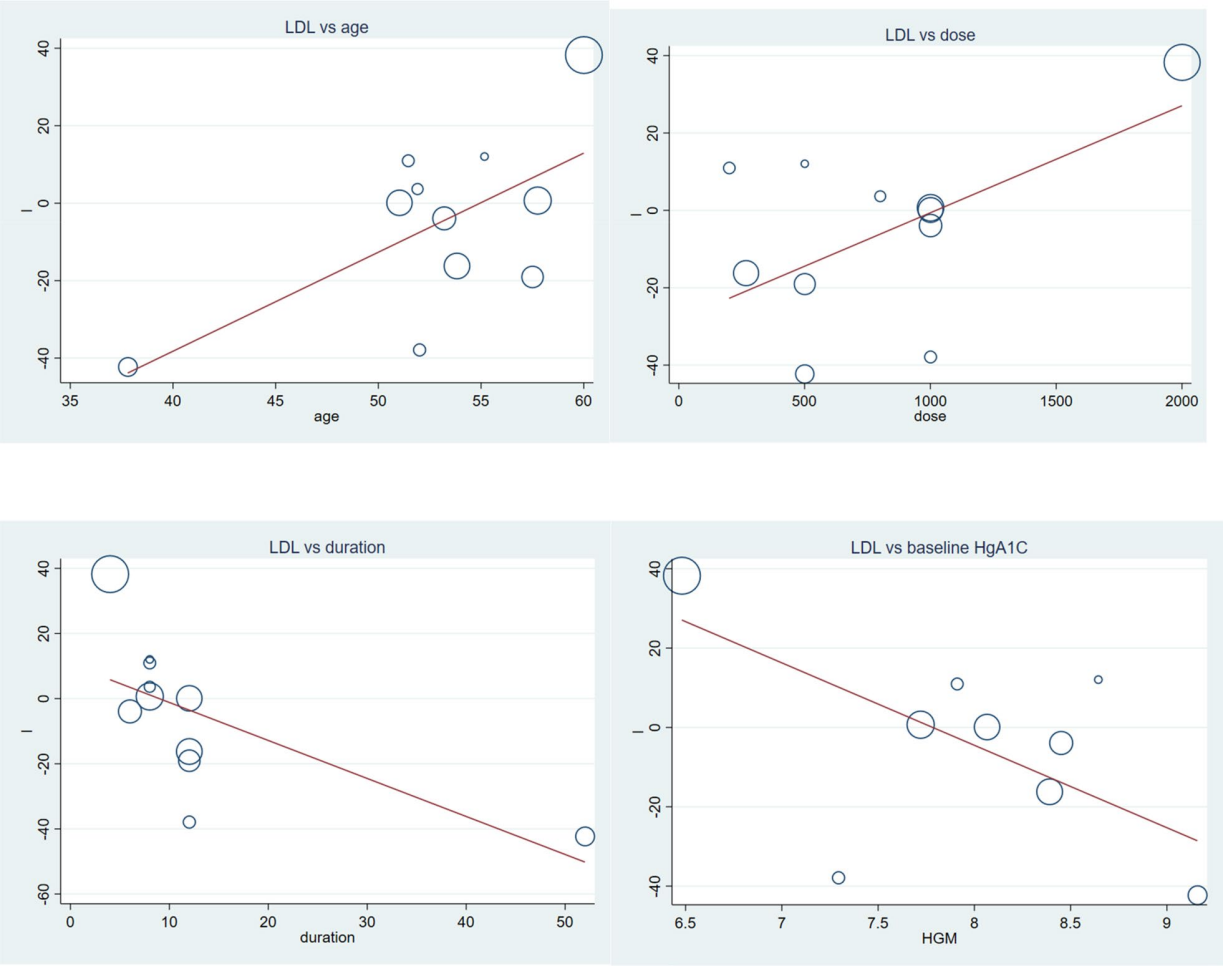
The association between LDL concentration measured as weighted mean difference and various factors. Younger age (β = 2.85 CI 0.29, 4.82: p-value = 0.031), lower dose (β = 0.02 CI: 0.00, 0.05: p-value = 0.039), longer duration (β = − 1.16 CI − 2.17, -0.16: p-value = 0.027) and higher baseline HgA1C (β = − 20.77 CI − 41.32, − 0.22: p-value = 0.048) were advantageous circumstances for vitamin C effect

Generally, vitamin C supplementation to diabetic patients didn’t reduce LDL level (Fig. 6). But, findings from subgroup analysis showed opposite effects; the lower the dose of vitamin C, the longer the duration of supplementation, or the younger the patients there were a significant reduction in LDL level. Higher dose, shorter duration and older age vitamin C supplementation significantly increased LDL level (Table 2). Meta-regression analysis of LDL and covariates (Table 3), showed mean age, dose of vitamin C, duration of supplementation and HgA1C were significant covariates. Vitamin C supplementation better works for younger patients, with smaller dose, longer period of treatment and higher HgA1C level (Fig. 7). In sensitivity analysis dropping a single study can change the effect size of the study, WMD = − 10.00 CI − 15.18, − 4.83 and there was a significant reduction of LDL level due to supplementation of vitamin C (Additional file 1: Fig. S3 and Table S3).

#### Vitamin C and HDL

**Fig. 8:**
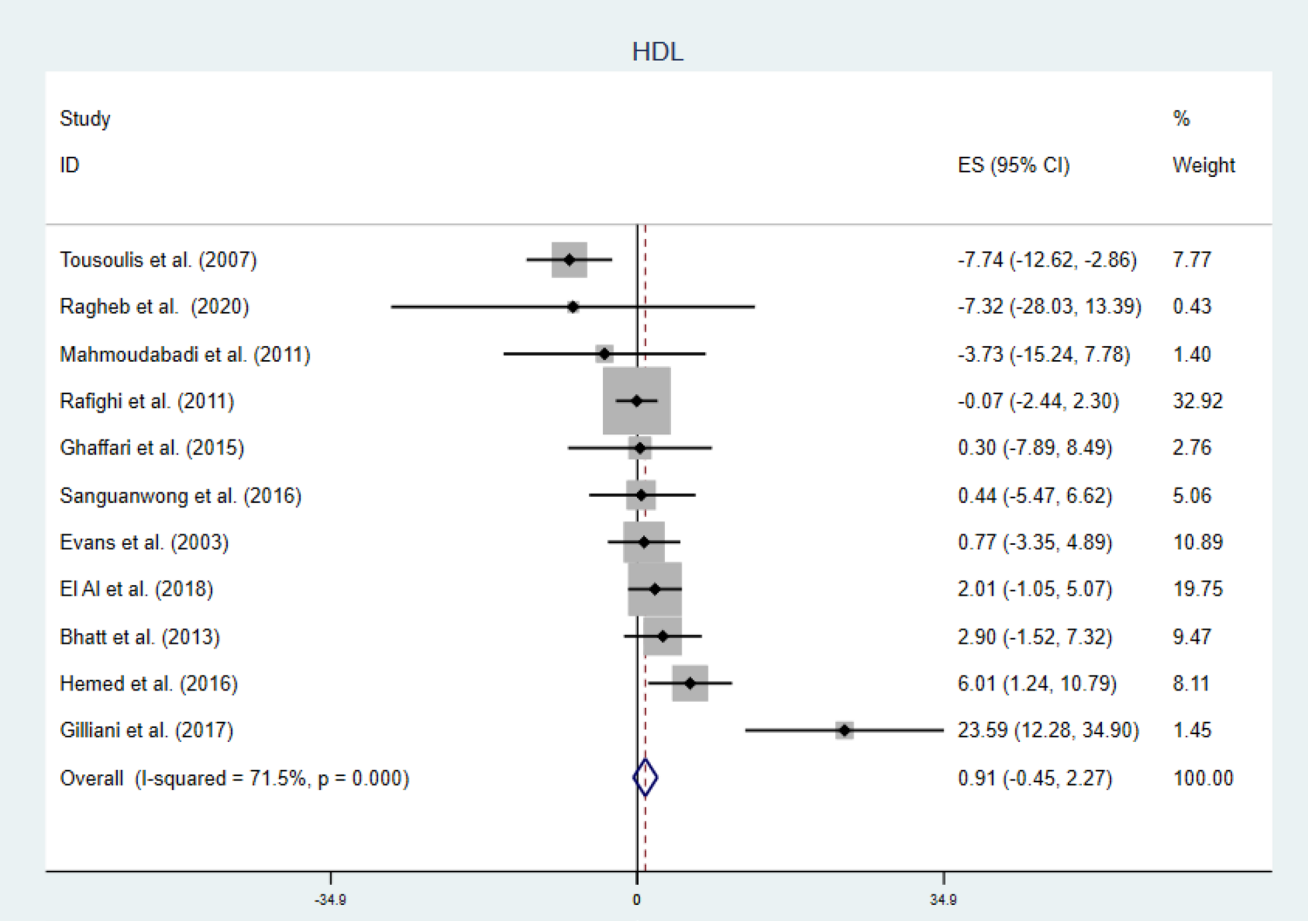
Forest plot showing no significant change in serum HDL in diabetic patients by vitamin C supplementation

**Fig. 9:**
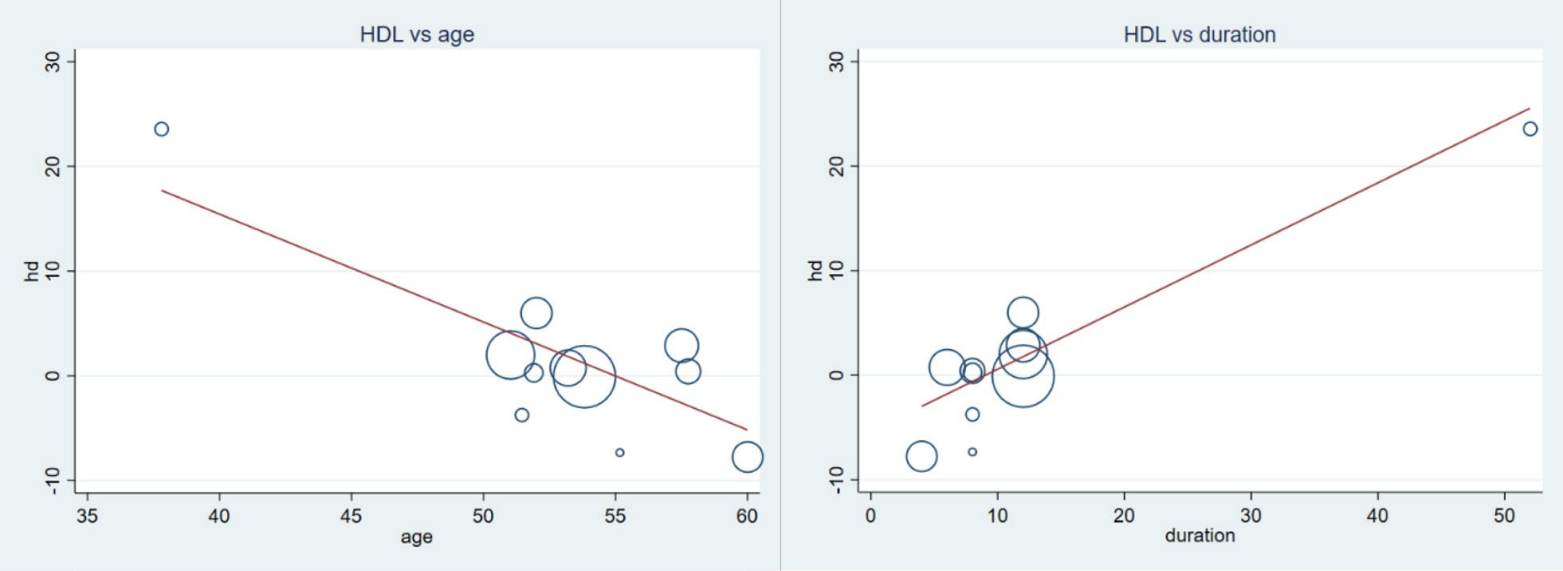
Meta-regression analysis showing the association of HDL concentration (WMD), and mean age and supplementation duration. Increment in HDL was associated with younger (β = − 1.03 CI − 1.63, − 0.42: p-value = 0.004) and longer duration (β = 0.59 CI 0.29, 0.89: p-value = 0.001) of treatment

Generally, vitamin C failed to increase serum HDL level WMD = 0.91 mg/dl (CI − 0.45, 2.27) p-value = 0.191 and I2 = 71.5% [forest plot given in Fig. 8]. Further subgroup analysis indicated longer duration of supplementation (WMD = 2.06 mg/dl, CI 0.45, 3.67 and p-value = 0.012) and younger patients (WMD = 3.53 mg/dl, CI 1.18, 5.88 and p-value = 0.003) may get beneficial effect from this vitamin (shown in Table 2). Moreover meta-regression analysis results showed baseline variables and dose have no influence on the response of patients regarding HDL. Younger age and longer duration of supplementation had better responses; (β = − 1.03, CI − 1.63, − 0.42 p-value = 0.004) and (β = 0.59 CI 0.29, 0.89 p-value = 0.001) respectively (Fig. 9). Sensitivity analysis detected one study which have much influence and elimination of this study showed the beneficial effect of vitamin C to boost HDL level [WMD = 1.63 CI 0.21, 3.05] as shown in Additional file 1: Fig. S4 and Table S4.

#### Secondary outcomes

**Fig. 10:**
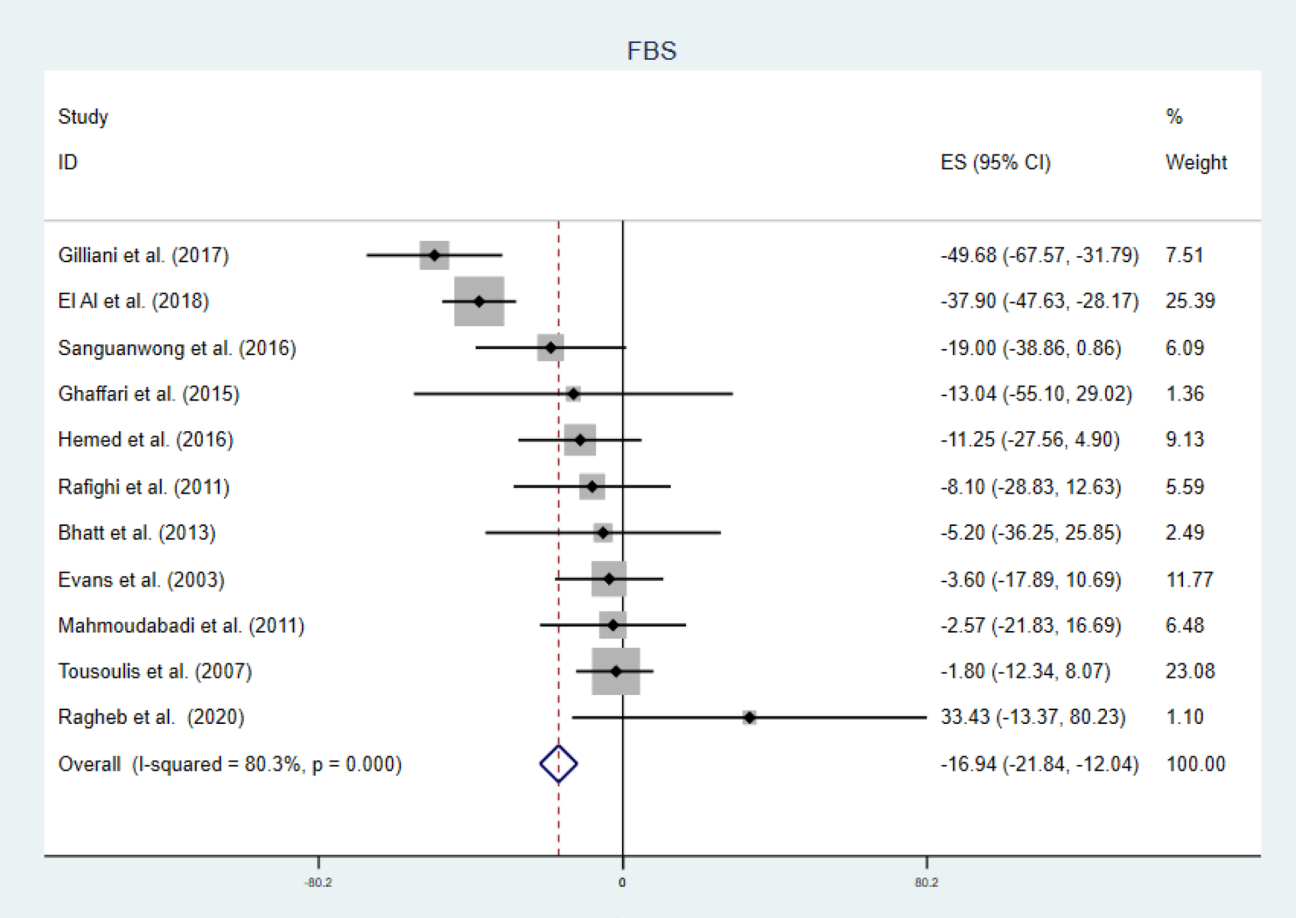
Forest plot of WMD of FBS, indicating significant reduction by vitamin C supplementation in type 2 DM

**Fig. 11:**
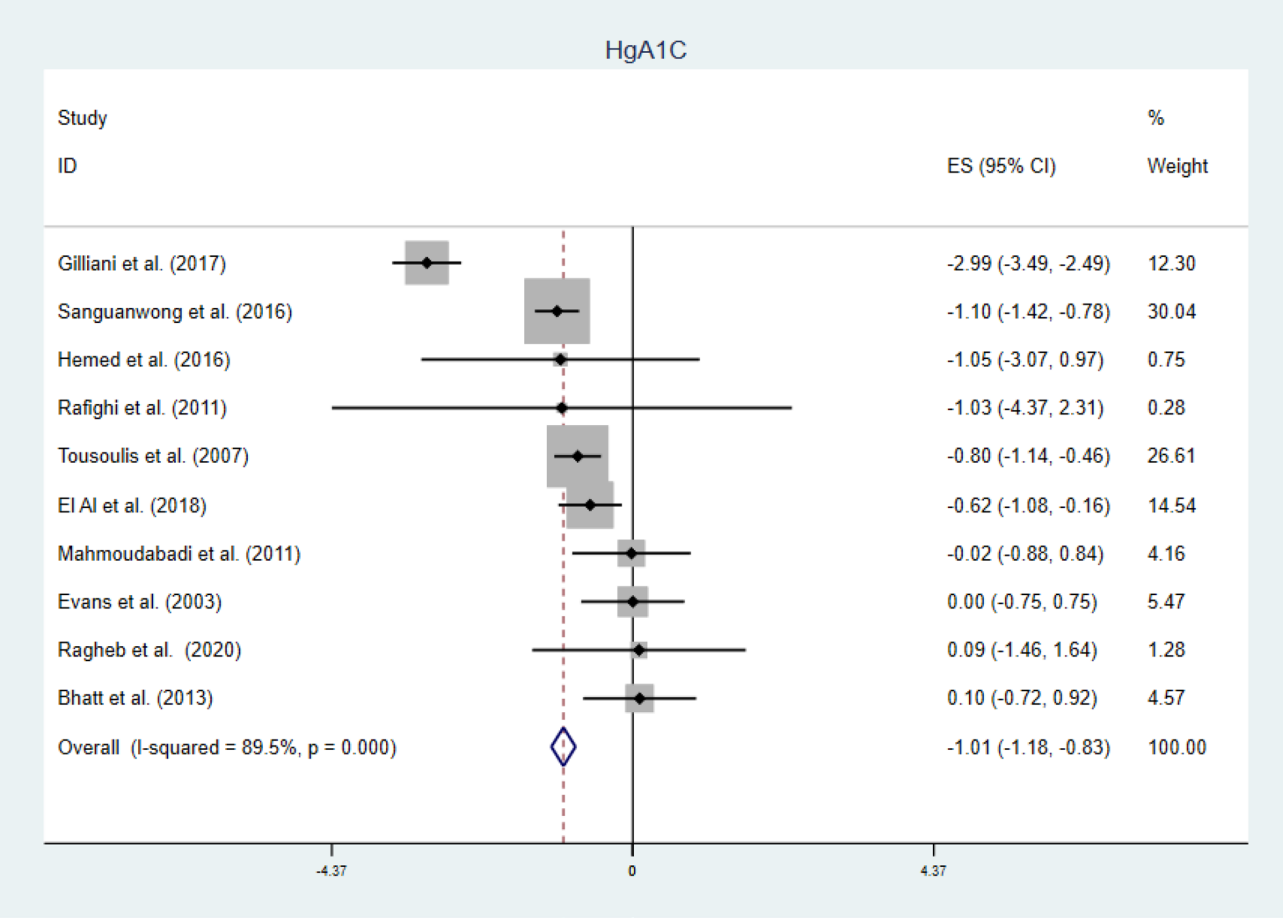
Forest plot of WMD of HgA1C (%), indicating significant reduction by vitamin C supplementation in type 2 DM

**Fig. 12:**
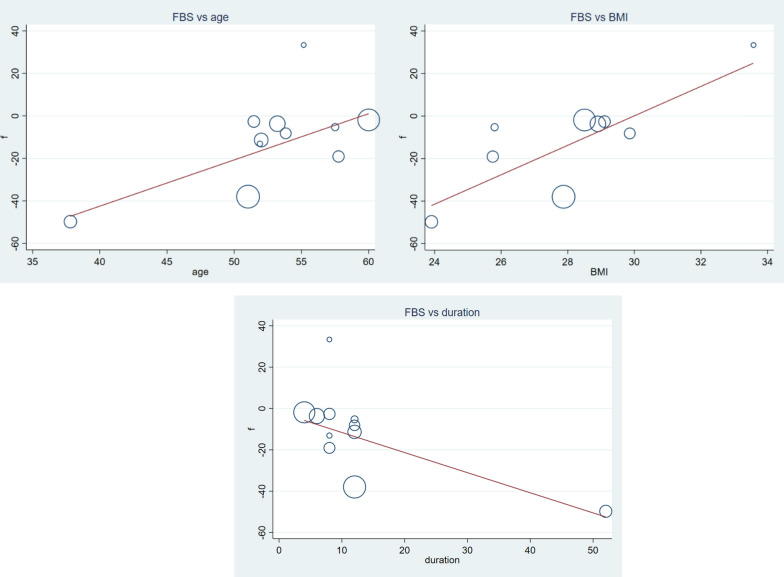
The association between FBS and covariates; age, duration of treatment and BMI. Reduction in FBS was associated with younger (β = 2.17 CI 0.47, 3.87: p-value = 0.018) and longer duration (β = − 0.97 CI − 1.73, − 0.21: p-value = 0.018) of treatment and lower BMI (β = 6.29 CI 1.23, 12.61: p-value = 0.024)

**Fig. 13:**
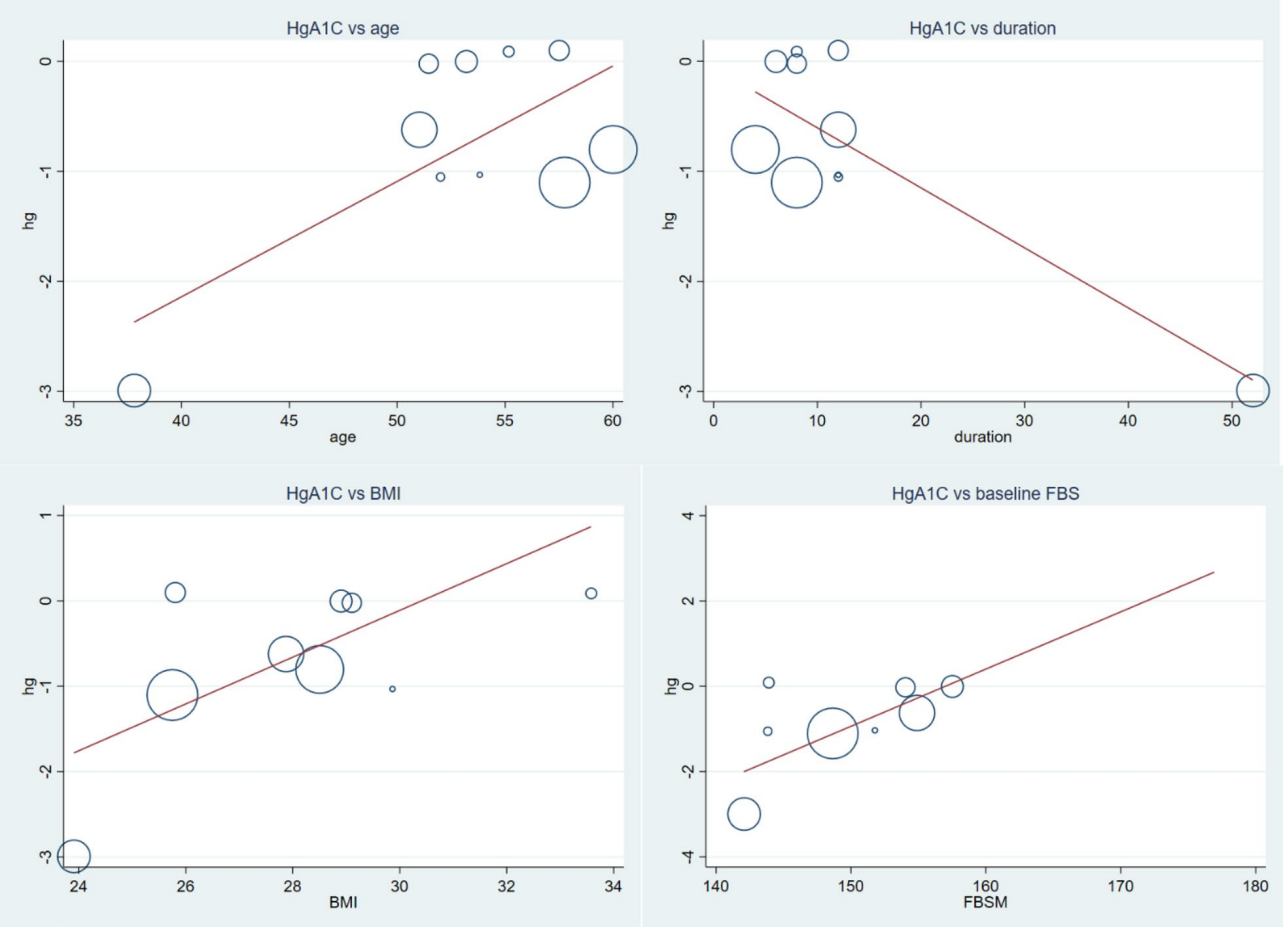
Meta-regression analysis showing the association of HgA1C concentration (WMD), and mean age, supplementation duration, BMI and baseline FBS. Decrement in HgA1C was associated with younger (β = 0.10 CI 0.01, 0.19: p-value = 0.032) and longer duration (β = − 0.05 CI − 0.08, − 0.02: p-value = 0.003) of treatment, lower BMI (β = 0.27 CI 0.00, 0.54: p-value = 0.049) and lower baseline FBS (β = 0.13 CI 0.005, 0.262: p-value = 0.044)

There were a significant reduction in FBS (Fig. 10) and HgA1C level (Fig. 11), (WMD = − 16.94 mg/dl CI − 21.84, − 12.04, p-value = 0.000 and I^2^ = 80.3%), and (WMD = − 1.01% CI − 1.18, − 0.83, p-value = 0.001 and I^2^ = 89.5%) respectively. Subgroup analysis for secondary outcomes revealed both FBS and HgA1C decreased significantly in both lower and higher doses. While we observe a significant reduction in HgA1C regardless of age and duration categorization, the FBS reduction was seen in longer duration of supplementation and younger patients (Table 2). In meta-regression analysis, age, duration and BMI was significant covariates for FBS (Fig. 12). Again, age, duration, baseline FBS and BMI were factors influencing HgA1C (Fig. 13). Diabetic patients having lower baseline FBS responds well for vitamin C supplementation. Reduction in FBS was associated with younger (β = 2.17 CI 0.47, 3.87: p-value = 0.018) and longer duration (β = − 0.97 CI − 1.73, − 0.21: p-value = 0.018) of treatment and lower BMI (β = 6.29 CI 1.23, 12.61: p-value = 0.024). Decrement in HgA1C was associated with younger (β = 0.10 CI 0.01, 0.19: p-value = 0.032) and longer duration (β = − 0.05 CI − 0.08, − 0.02: p-value = 0.003) of treatment, lower BMI (β = 0.27 CI 0.00, 0.54: p-value = 0.049) and lower baseline FBS (β = 0.13 CI 0.005, 0.262: p-value = 0.044). Sensitivity analysis for detection of robustness was conducted for both secondary outcomes, given in Additional file 1: Figs. S5 & S6 and Tables S5 & S6.

#### Publication bias

Visual inspection of funnel plot for all outcomes showed no asymmetry, given in (Additional file 1: Figs. S7–S12). Egger’s regression test also indicated no significant publication bias for all outcomes (Additional file 1: Table S7).

## Discussion

Vitamin C supplementation had no significant effect on TC, LDL and HDL. However, it reduced TG and secondary outcomes (FBS and HgA1C). Subgroup analysis also depicted younger patients, longer duration of treatment and higher dose were important factors in response of vitamin C supplementation. Despite insufficient evidence for lipid lowering capacity of vitamin C supplementation in T2DM patients, this meta-analysis gave a clue about specific group of population that might be benefited from this vitamin. Specifically, younger diabetic patients were responsive for the supplementation and likely to be benefited. In addition, meta-regression analysis indicated the significant role of patient age, duration of treatment, supplementation dose, BMI and other baseline variables.

A systematic review and meta-analysis previously showed insignificant role of vitamin C on lipid profile of patients including diabetic and healthy individuals generally [36]. With the same scenario, the former study also demonstrated specific groups of population benefited from vitamin C, especially lower baseline vitamin C status and individuals with higher risk of cardiovascular diseases. McRae in 2008 noted a significant reduction in serum LDL and TG without statistically significant increment in HDL [37]. Although, there are differences in the study populations, there is no concrete previous evidence both to support or rule out the role of vitamin C in management of lipid abnormalities.

In T2DM, exaggerated free radical activity and lipid peroxidation have been demonstrated, such enhanced oxidative stress was associated with poor glycemic control and appearance other metabolic complications including dyslipidemias. Vitamin C, a known anti-oxidant agent, exert its protective effect by scavenging free radicals [38, 39]. It also presents in lower plasma and intracellular concentrations in diabetic patients than healthy controls [40]. Previous researches indicated daily vitamin C supplementation protects against oxidative stress in diabetic patients, macro and microangiopathy, and improve metabolic control [41]. These metabolic benefits could interfere with the lipid metabolic pathway and could reduce diabetic associated dyslipidemias.

In all four primary outcomes, the positive effects of vitamin C supplementation was witnessed in younger patients. Along with the age related pharmacokinetic and pharmacodynamic changes of this water soluble vitamin [42], one explanation could be age related difference in baseline vitamin C concentration. This meta-analysis doesn’t measure the role of baseline vitamin C concentration or plasma vitamin C concentration due to insufficient data from original studies. Regarding entry to trial vitamin C concentration, older individuals have lower plasma vitamin C compared with younger patients [43]. Also younger individuals may have ascorbic acid recycling capacity to balance their redox system and to respond to the supplementation [44]. The other possible reason might be less efficient absorption in older individuals [45]. Contrary to the notion that lower baseline vitamin C in older patients could contribute to the no effect is the finding of A.W. Ashor et al. [36], indicated individuals with lower baseline vitamin C responds well. It should also be noted that the younger and older division of the population in this study is arbitrary, merely based on participants mean age. The duration of treatment might provide a clue to reach optimum plasma concentration of vitamin C for physiologic effects.

The intestinal absorption of vitamin C is a saturable, dose dependent mechanism mainly by sodium dependent active transport systems [46]. It is also suggested that higher dose of vitamin C is necessary to maintain normal plasma concentration of this substance in critically ill individuals [47]. The response of diabetic patients to higher dose of vitamin C seems to be associated with the intestinal availability and the normal plasma concentration. Despite the deficiency of this meta-analysis in failing to consider plasma concentration of vitamin C, previous researches suggested the crucial impact of plasma vitamin C concentration in its physiologic effect [48].

Although it is well established that vitamin C supplementation reduces FBS and HgA1C, the reduction effect on TG is not settled. In systematic review and meta-analysis [48], vitamin C had crucial role for glycemic control especially in patients with diabetes.

Apart from quality (risk of bias given in the Additional file 1) and limitations from the primary studies, this meta-analysis had certain drawbacks. First, the baseline plasma vitamin C or concentration vitamin C at the end was not evaluated. Reports indicated the influence of plasma concentration [besides the dose of supplementation] on physiologic effects, readers should bear in mind that the reported results were not related with plasma concentration of vitamin C at baseline or final. While the patients are diabetic, they are on different anti-diabetic drugs. It is worth mentioning that different drugs may have influence in vitamin C effects, this research didn’t consider specific diabetic drugs and the duration of treatment of these drugs. Other conditions that might alter the results of this research were the duration of diabetes and plasma insulin level. All these factors were not evaluated due inconsistent report or absence in the original studies.

## Conclusion

There is no sufficient evidence to support vitamin C supplementation for dyslipidemias in T2DM patients. Specific group of patients might have benefited including younger diabetic patients. Future researches should give emphasis on the duration of treatment, the dose of vitamin C and baseline values.

## Supplementary Information

**Additional file 1.** **Table S1** Sensitivity analysis of the effect of vitamin C on cholesterol in diabetic patients, indicates dropping Tousoulis et al. [35] gives significant reduction in cholesterol level. **Figure S1** Cholesterol. **Table S2** Sensitivity analysis for triglycerides. **Figure S2** TG. **Table S3** Sensitivity analysis for LDL, shows the effect of vitamin C in LDL reduction by omitting Tousoulis et al. (2007). **Figure S3** LDL. **Table S4** Sensitivity of analysis for HDL, no single study was influencing the outcome. **Figure S4** HDL. **Table S5** Sensitivity analysis for FBS for detection of robustness of study. **Figure S5** FBS. **Table S6** Sensitivity of analysis for HgA1C, no single study was influencing the outcome. **Figure S7** Funnel plot for cholesterol, showing no asymmetry. **Figure S8** Funnel plot for triglycerides, showing no asymmetry. **Figure S9** Funnel plot for LDL, showing no asymmetry. **Figure S10** Funnel plot for HDL, showing no asymmetry. **Figure S11** Funnel plot for FBS, showing no visual asymmetry. **Figure S12** Funnel plot for HgA1C, showing no asymmetry. **Table S7** Publication bias, indicates no significant publication bias for each outcome. **Table S2** Risk of bias for included studies.

## Data Availability

The datasets used and/or analyzed during the current study are available from the corresponding author on reasonable request.

## Abbreviations

BMI: Body mass index
FBS: Fasting blood sugar
HDL: High density lipoprotein
HgA1C: Hemoglobin A1C
LDL: Low density lipoprotein
PRISMA: Prefered reporting items for systematic reviews and meta-analysis
T2DM: Type 2 Diabetes Mellitus
TC: Total cholesterol
TG: Total triglycerides
WMD: Weighted mean difference

## References

[1] Howard BV (1999). Insulin resistance and lipid metabolism. Am J Cardiol.

[2] Steinberger J, Moorehead C, Katch V, Rocchini AP (1995). Relationship between insulin resistance and abnormal lipid profile in obese adolescents. J Pediatr.

[3] Suryawanshi NP, Bhutey AK, Nagdeote AN, Jadhav AA, Manoorkar GS (2006). Study of lipid peroxide and lipid profile in diabetes mellitus. Indian J Clin Biochem.

[4] Reaven GM, Chen YD, Jeppesen J, Maheux P, Krauss RM (1993). Insulin resistance and hyperinsulinemia in individuals with small, dense low density lipoprotein particles. J Clin Invest.

[5] Katz LEL, Bacha F, Gidding SS, Weinstock RS, Ghormli LE, Libman I (2018). Lipid profiles, inflammatory markers, and insulin therapy in youth with type 2 diabetes. J Pediatr.

[6] Mooradian AD (2009). Dyslipidemia in type 2 diabetes mellitus. Nat Clin Pract Endocrinol Metab.

[7] Mooradian AD, Haas MJ, Wehmeier KR, Wong NCW (2008). Obesity-related changes in high-density lipoprotein metabolism. Obesity (Silver Spring).

[8] Mooradian AD, Albert SG, Haas MJ (2007). Low serum high-density lipoprotein cholesterol in obese subjects with normal serum triglycerides: the role of insulin resistance and inflammatory cytokines. Diabetes Obes Metab.

[9] U Kolhar, P Priyanka. Study of serum lipid profile in type 2 diabetes mellitus patients and its association with diabetic nephropathy. Int J Adv Med 2017;4:1513-6. https://doi.org/10.18203/2349-3933.ijam20174639.

[10] Bonnet F, Cooper ME. Potential influence of lipids in diabetic nephropathy: insights from experimental data and clinical studies. /data/revues/12623636/00260004/254/ 2008. 11011217

[11] Gall MA, Hougaard P, Borch-Johnsen K, Parving HH (1997). Risk factors for development of incipient and overt diabetic nephropathy in patients with non-insulin dependent diabetes mellitus: prospective, observational study. BMJ.

[12] Obermayr RP, Temml C, Knechtelsdorfer M, Gutjahr G, Kletzmayr J, Heiss S (2008). Predictors of new-onset decline in kidney function in a general middle-european population. Nephrol Dial Transplant.

[13] Papavasileiou E, Davoudi S, Roohipoor R, Cho H, Kudrimoti S, Hancock H (2017). Association of serum lipid levels with retinal hard exudate area in African Americans with type 2 diabetes. Graefes Arch Clin Exp Ophthalmol.

[14] Rajbhandari S, Hamid FS, Harris N, Tesfaye S (2018). Lipid profile as a predictor of Neuropathy: the Sheffield Prospective Diabetes Study. J Diab Endocrinol Assoc Nepal.

[15] Virally M, Blicklé J-F, Girard J, Halimi S, Simon D, Guillausseau P-J. Type 2 diabetes mellitus: epidemiology, pathophysiology, unmet needs and therapeutical perspectives. /data/revues/12623636/00330004/231/ 2008. 10.1016/j.diabet.2007.07.001 17703979

[16] Carr AC, Frei B (1999). Toward a new recommended dietary allowance for vitamin C based on antioxidant and health effects in humans. Am J Clin Nutr.

[17] Halliwell B (1996). Vitamin C: antioxidant or pro-oxidant in vivo?. Free Radic Res.

[18] Podmore ID, Griffiths HR, Herbert KE, Mistry N, Mistry P, Lunec J (1998). Vitamin C exhibits pro-oxidant properties. Nature.

[19] Golbidi S, Ebadi SA, Laher I (2011). Antioxidants in the treatment of diabetes. Curr Diabetes Rev.

[20] Lee D-H, Folsom AR, Harnack L, Halliwell B, Jacobs DR (2004). Does supplemental vitamin C increase cardiovascular disease risk in women with diabetes?. Am J Clin Nutr.

[21] Liberati A, Altman DG, Tetzlaff J, Mulrow C, Gøtzsche PC, Ioannidis JPA (2009). The PRISMA statement for reporting systematic reviews and meta-analyses of studies that evaluate health care interventions: explanation and elaboration. PLOS Med.

[22] Higgins JPT, Altman DG, Gøtzsche PC, Jüni P, Moher D, Oxman AD (2011). The Cochrane Collaboration’s tool for assessing risk of bias in randomised trials. BMJ.

[23] Higgins JPT, Thompson SG, Deeks JJ, Altman DG (2003). Measuring inconsistency in meta-analyses. BMJ.

[24] Wan X, Wang W, Liu J, Tong T (2014). Estimating the sample mean and standard deviation from the sample size, median, range and/or interquartile range. BMC Med Res Methodol.

[25] Bhatt JK, Thomas S, Nanjan MJ (2012). Effect of oral supplementation of vitamin C on glycemic control and lipid profile in patients with type 2 diabetes mellitus. Int J Pharm Pharm Sci.

[26] El-Aal AA, El-Ghffar EAA, Ghali AA, Zughbur MR, Sirdah MM (2018). The effect of vitamin C and/or E supplementations on type 2 diabetic adult males under metformin treatment: a single-blinded randomized controlled clinical trial. Diab Metab Syndr Clin Res Rev.

[27] Evans M, Anderson RA, Smith JC, Khan N, Graham JM, Thomas AW (2003). Effects of insulin lispro and chronic vitamin C therapy on postprandial lipaemia, oxidative stress and endothelial function in patients with type 2 diabetes mellitus. Eur J Clin Invest.

[28] Ghaffari P, Nadiri M, Gharib A, Rahimi F (2015). The effects of vitamin C on diabetic patients. Der Pharmacia Lettre.

[29] Gillani SW, Sulaiman SAS, Abdul MIM, Baig MR (2017). Combined effect of metformin with ascorbic acid versus acetyl salicylic acid on diabetes-related cardiovascular complication; a 12-month single blind multicenter randomized control trial. Cardiovasc Diabetol.

[30] Hamed A, Zinati SMA, Swirky AA (2016). The effect of vitamin C alone or in combination with vitamin E on fasting blood glucose, glycosylated hemoglobin and lipid profile in type 2 diabetic patients (Gaza Strip). Jordan J Pharmaceutical Sci.

[31] Mahmoudabadi MMS, Djalali M, Djazayery SA, Keshavarz SA, Eshraghian MR, Yaraghi AAS (2011). Effects of eicosapentaenoic acid and vitamin C on glycemic indices, blood pressure, and serum lipids in type 2 diabetic Iranian males. J Res Med Sci.

[32] Rafighi Z, Arab S, Yusof RM, Shiva A (2011). The effect of vitamin C and E on lipid profile in type 2 diabetes mellitus patients. Glob J Health Sci.

[33] Ragheb SR, El Wakeel LM, Nasr MS, Sabri NA (2020). Impact of Rutin and Vitamin C combination on oxidative stress and glycemic control in patients with type 2 diabetes. Clin Nutr ESPEN.

[34] Sanguanwong S, Tangvarasittichai O, Sengsuk C, Tangvarasittichai S (2016). Oral supplementation of vitamin C reduced lipid peroxidation and insulin resistance in patients with type 2 diabetes mellitus. Int J Toxicol Pharmacol Res.

[35] Tousoulis D, Antoniades C, Vasiliadou C, Kourtellaris P, Koniari K, Marinou K (2007). Effects of atorvastatin and vitamin C on forearm hyperaemic blood flow, asymmentrical dimethylarginine levels and the inflammatory process in patients with type 2 diabetes mellitus. Heart.

[36] Ashor AW, Siervo M, van der Velde F, Willis ND, Mathers JC (2016). Systematic review and meta-analysis of randomised controlled trials testing the effects of vitamin C supplementation on blood lipids. Clin Nutr.

[37] McRae MP (2008). Vitamin C supplementation lowers serum low-density lipoprotein cholesterol and triglycerides: a meta-analysis of 13 randomized controlled trials. J Chiropractic Med.

[38] Collier A, Wilson R, Bradley H, Thomson JA, Small M (1990). Free radical activity in type 2 diabetes. Diabet Med.

[39] Dd W, Gw B, Ku I (1986). The antioxidant efficiency of vitamin C is concentration-dependent. Biochim Biophys Acta.

[40] Stankova L, Riddle M, Larned J, Burry K, Menashe D, Hart J (1984). Plasma ascorbate concentrations and blood cell dehydroascorbate transport in patients with diabetes mellitus. Metabolism.

[41] Paolisso G, Balbi V, Volpe C, Varricchio G, Gambardella A, Saccomanno F (1995). Metabolic benefits deriving from chronic vitamin C supplementation in aged non-insulin dependent diabetics. J Am Coll Nutr.

[42] Mangoni AA, Jackson SHD (2004). Age-related changes in pharmacokinetics and pharmacodynamics: basic principles and practical applications. Br J Clin Pharmacol.

[43] Birlouez-Aragon I, Delcourt C, Tessier F, Papoz L, POLA Study Group (2001). Associations of age, smoking habits and diabetes with plasma vitamin C of elderly of the POLA study. Int J Vitamin Nutr Res.

[44] Lykkesfeldt J (2002). Increased oxidative damage in vitamin C deficiency is accompanied by induction of ascorbic acid recycling capacity in young but not mature guinea pigs. Free Radical Res.

[45] van der Loo B, Bachschmid M, Spitzer V, Brey L, Ullrich V, Lüscher TF (2003). Decreased plasma and tissue levels of vitamin C in a rat model of aging: implications for antioxidative defense. Biochem Biophys Res Commun.

[46] Lykkesfeldt J, Tveden-Nyborg P (2019). The pharmacokinetics of vitamin C. Nutrients.

[47] de Grooth H-J, Manubulu-Choo W-P, Zandvliet AS, Spoelstra-de-Man AME, Girbes AR, Swart EL (2018). Vitamin C pharmacokinetics in critically ill patients: a randomized trial of four IV regimens. Chest.

[48] Ashor AW, Werner AD, Lara J, Willis ND, Mathers JC, Siervo M (2017). Effects of vitamin C supplementation on glycaemic control: a systematic review and meta-analysis of randomised controlled trials. Eur J Clin Nutr.

