# Supplementary material for: The effect of vitamin C supplementation on lipid profile of type 2 diabetic patients: a systematic review and meta-analysis of clinical trials
Source: Diabetol Metab Syndr. 2021 Mar 2;13:24. doi: 10.1186/s13098-021-00640-9 (PMC7923652; doi:10.1186/s13098-021-00640-9)
Supplement: Supplementary file 1 — Additional file 1. Table S1 Sensitivity analysis of the effect of vitamin C on cholesterol in diabetic patients, indicates dropping Tousoulis et al. [35] gives significant reduction in cholesterol level. Figure S1 Cholesterol. Table S2 Sensitivity analysis for triglycerides. Figure S2 TG. Table S3 Sensitivity analysis for LDL, shows the effect of vitamin C in LDL reduction by omitting Tousoulis et al. (2007). Figure S3 LDL. Table S4 Sensitivity of analysis for HDL, no single study was influencing the outcome. Figure S4 HDL. Table S5 Sensitivity analysis for FBS for detection of robustness of study. Figure S5 FBS. Table S6 Sensitivity of analysis for HgA1C, no single study was influencing the outcome. Figure S7 Funnel plot for cholesterol, showing no asymmetry. Figure S8 Funnel plot for triglycerides, showing no asymmetry. Figure S9 Funnel plot for LDL, showing no asymmetry. Figure S10 Funnel plot for HDL, showing no asymmetry. Figure S11 Funnel plot for FBS, showing no visual asymmetry. Figure S12 Funnel plot for HgA1C, showing no asymmetry. Table S7 Publication bias, indicates no significant publication bias for each outcome. Table S2 Risk of bias for included studies. [file 13098_2021_640_MOESM1_ESM.docx]

Supplementary files

sTable 1: sensitivity analysis of the effect of vitamin C on cholesterol in diabetic patients, indicates dropping Tousoulis et al. (2007) gives significant reduction in cholesterol level.

| Cholesterol | | |
| --- | --- | --- |
| Study omitted | Estimate | 95% CI |
| Bhatt et al. (2013) | -1.9529537 | -8.2746601, 4.368753 |
| El Al et al. (2018) | -3.8367167 | -10.313442, 2.6400087 |
| Evans et al. (2003) | -4.3871102 | -10.484113 1.7098917 |
| Ghaffari et al. (2015) | -4.4821191 | -10.461729 1.4974906 |
| Gilliani et al. (2017) | -2.8803804 | -8.8465776 3.0858173 |
| Hemed et al. (2016) | -2.7656302 | -8.8138294 3.2825689 |
| Mahmoudabadi et al. (2011) | -5.0695972 | -11.148505 1.0093101 |
| Rafighi et al. (2011) | -4.9929743 | -11.146329 1.1603808 |
| Ragheb et al. (2020) | -4.860096 | -10.785718 1.0655266 |
| Sanguanwong et al. (2016) | -3.6411419 | -10.254542 2.9722581 |
| Tousoulis et al. (2007) | -9.0607414 | -15.321947 -2.7995358 |
| Combined | -4.3597307 | -10.237165 1.5177034 |


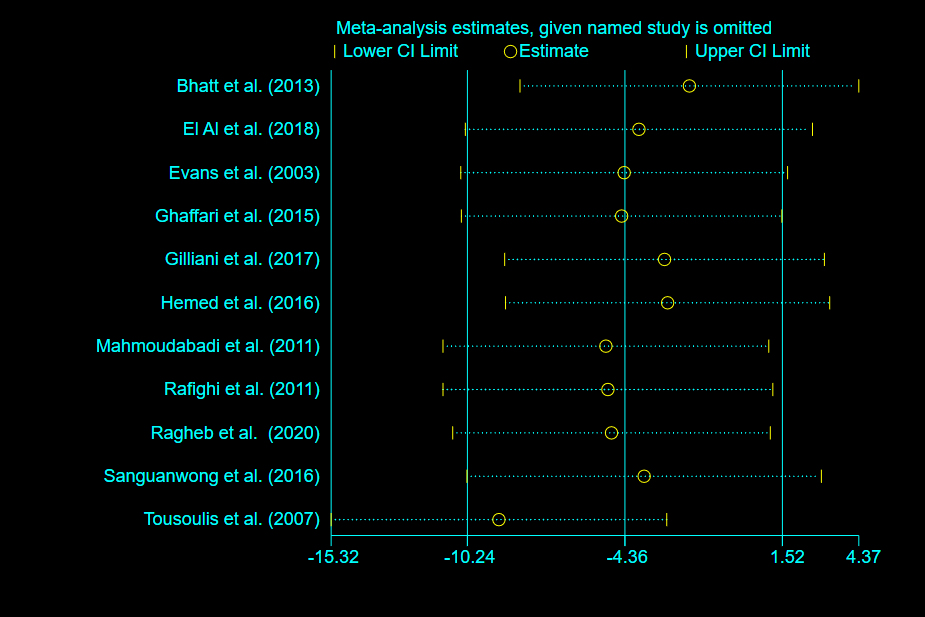


sFigure 1: cholesterol

sTable 2: sensitivity analysis for triglycerides

| Triglyceride | | |
| --- | --- | --- |
| Study omitted | Estimate | [95% Conf. Interval] |
| Bhatt et al. (2013) | -11.220135 | -23.057226, 0.61695611 |
| El Al et al. (2018) | -6.0272446 | -17.38204, 5.3275509 |
| Evans et al. (2003) | -15.55821 | -27.085886, -4.0305352 |
| Ghaffari et al. (2015) | -11.148767 | -21.583357, -0.71417773 |
| Gilliani et al. (2017) | -6.0557027 | -16.907898, 4.7964921 |
| Hemed et al. (2016) | -10.31107 | -21.030983, 0.40884116 |
| Mahmoudabadi et al. (2011) | -12.139776. | -22.720032, -1.5595206 |
| Rafighi et al. (2011) | -10.012051 | -20.790028, 0.76592749 |
| Ragheb et al. (2020) | -18.73148 | -29.3983, -8.0646601 |
| Sanguanwong et al. (2016) | -9.6665478 | -21.088135, 1.7550405 |
| Tousoulis et al. (2007) | -11.393877 | -21.904495, -0.88325703 |
| Combined | -11.148768 | -21.583357, -0.71417771 |


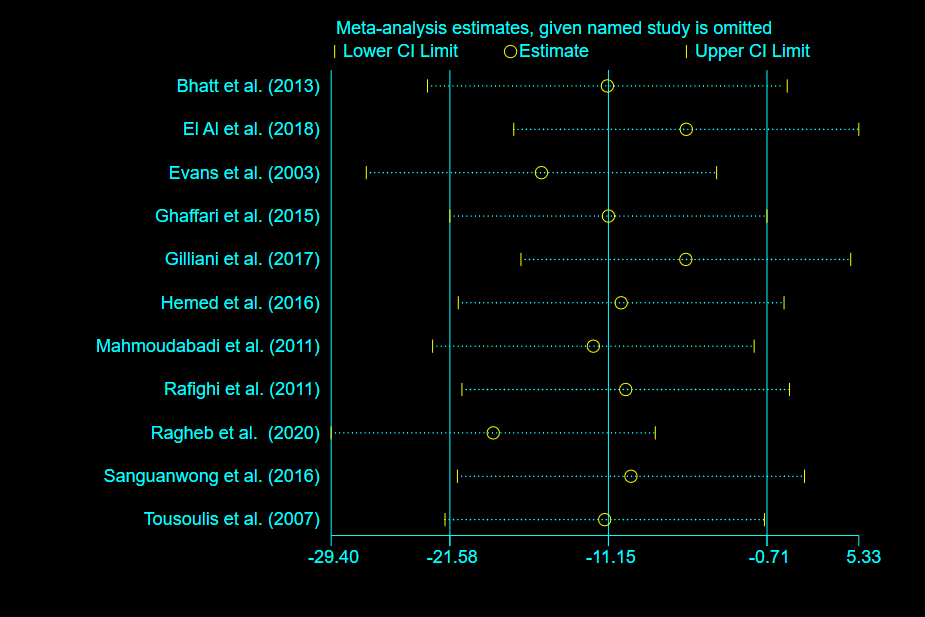


sFigure 2 TG

sTable 3: sensitivity analysis for LDL, shows the effect of vitamin C in LDL reduction by omitting Tousoulis et al. (2007)

| LDL | | |
| --- | --- | --- |
| Study omitted | Estimate | [95% Conf. Interval] |
| Bhatt et al. (2013) | 4.826314 | .17517252, 9.4774551 |
| El Al et al. (2018) | 3.0923951 | -1.6542051 7.838995 |
| Evans et al. (2003) | 3.4679098 | -1.2165761 8.1523962 |
| Ghaffari et al. (2015) | 2.7033122 | -1.7900449 7.1966696 |
| Gilliani et al. (2017) | 5.9514842 | 1.3535591 10.549409 |
| Hemed et al. (2016) | 3.8651021 | -.6382305 8.3684349 |
| Mahmoudabadi et al. (2011) | 2.5069156 | -1.9929931 7.0068245 |
| Rafighi et al. (2011) | 5.48171 | .72746193 10.235958 |
| Ragheb et al. (2020) | 2.6291547 | -1.8352375 7.0935473 |
| Sanguanwong et al. (2016) | 3.0570045 | -1.738322 7.8523307 |
| Tousoulis et al. (2007) | -10.006846 | -15.182658 -4.8310337 |
| Combined | 2.7262139 | -1.715219 7.1676474 |


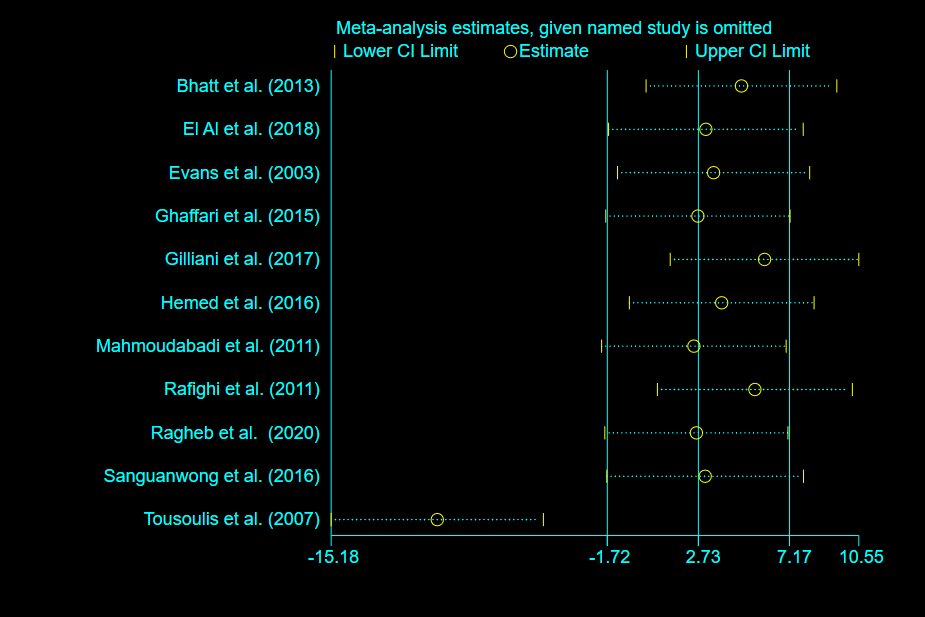


sFigure 3 LDL

sTable 4: sensitivity of analysis for HDL, no single study was influencing the outcome.

| HDL | | |
| --- | --- | --- |
| Study omitted | Estimate | [95% Conf. Interval] |
| Bhatt et al. (2013) | .69826603 | -.73094082 2.1274729 |
| El Al et al. (2018) | .63514978 | -.88287187 2.1531713 |
| Evans et al. (2003) | .9233892 | -.51722991 2.3640084 |
| Ghaffari et al. (2015) | .92387843 | -.45514664 2.3029034 |
| Gilliani et al. (2017) | .57393545 | -.79588479 1.9437557 |
| Hemed et al. (2016) | .45623109 | -.96239775 1.8748599 |
| Mahmoudabadi et al. (2011) | .97231752 | -.39715675 2.3417916 |
| Rafighi et al. (2011) | 1.3860662 | -.27434704 3.0464795 |
| Ragheb et al. (2020) | .94230229 | -.42052138 2.305126 |
| Sanguanwong et al. (2016) | .93155426 | -.46410161 2.3272102 |
| Tousoulis et al. (2007) | 1.6346564 | .21868531 3.0506277 |
| Combined | .9066782 | -.45320427 2.2665606 |


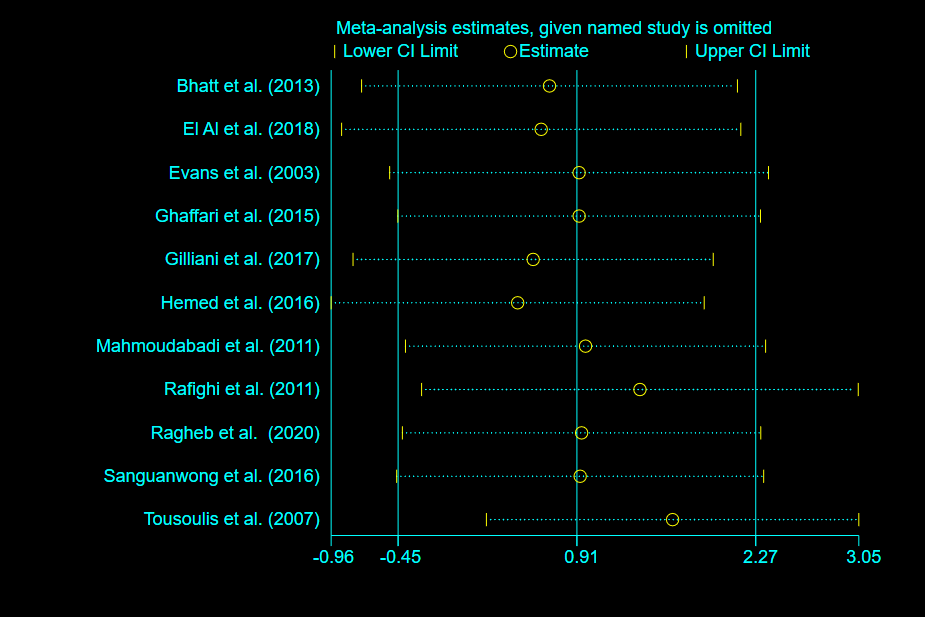


sFigure 4 HDL

sTable 5: sensitivity analysis for FBS for detection of robustness of study

| FBS | | |
| --- | --- | --- |
| Study omitted | Estimate | [95% Conf. Interval] |
| Bhatt et al. (2013) | -17.238173 | -22.20336 -12.272985 |
| El Al et al. (2018) | -9.8042183 | -15.480412 -4.1280251 |
| Evans et al. (2003) | -18.71763 | -23.93737 -13.497889 |
| Ghaffari et al. (2015) | -16.991716 | -21.928268 -12.055165 |
| Gilliani et al. (2017) | -14.27914 | -19.377228 -9.1810541 |
| Hemed et al. (2016) | -17.50922 | -22.652409 -12.366034 |
| Mahmoudabadi et al. (2011) | -17.933624 | -23.003544 -12.863704 |
| Rafighi et al. (2011) | -17.461695 | -22.507755 -12.415633 |
| Ragheb et al. (2020) | -17.496956 | -22.426981 -12.56693 |
| Sanguanwong et al. (2016) | -16.804193 | -21.863693 -11.744695 |
| Tousoulis et al. (2007) | -21.480806 | -27.071167 -15.890449 |
| Combined | -16.938021 | -21.840918 -12.035123 |


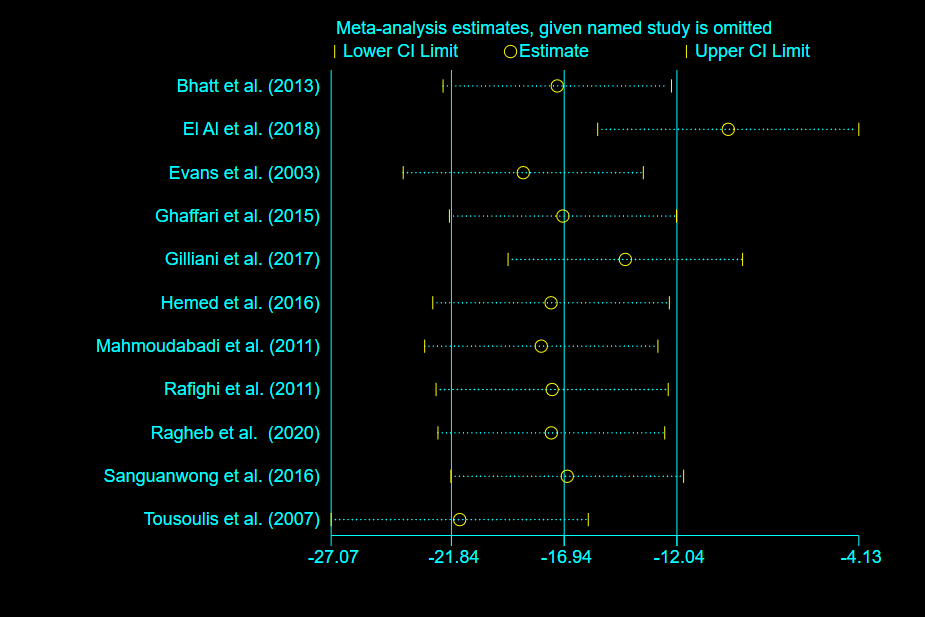


sFigure 5 FBS

sTable 6: sensitivity of analysis for HgA1C, no single study was influencing the outcome.

| HgA1C | | |
| --- | --- | --- |
| Study omitted | Estimate | [95% Conf. Interval] |
| Bhatt et al. (2013) | -1.0602475 | -1.2397866 -.88070834 |
| El Al et al. (2018) | -1.0730257 | -1.2627406 -.88331085 |
| Evans et al. (2003) | -1.0654327 | -1.2458186 -.88504678 |
| Ghaffari et al. (2015) | -1.0071707 | -1.1825551 -.83178616 |
| Gilliani et al. (2017) | -.72897696 | -.91626108 -.54169285 |
| Hemed et al. (2016) | -1.0068454 | -1.1828946 -.830796 |
| Mahmoudabadi et al. (2011) | -1.0500083. | -1.2291577 -.87085891 |
| Rafighi et al. (2011) | -1.0071075 | -1.1827343 -.83148074 |
| Ragheb et al. (2020) | -1.0214001 | -1.1979182 -.84488201 |
| Sanguanwong et al. (2016) | -.96731311 | -1.1769958 -.75763047 |
| Tousoulis et al. (2007) | -1.0822824 | -1.2870065 -.87755835 |
| Combined | -1.0071706 | -1.1825551 -.83178617 |


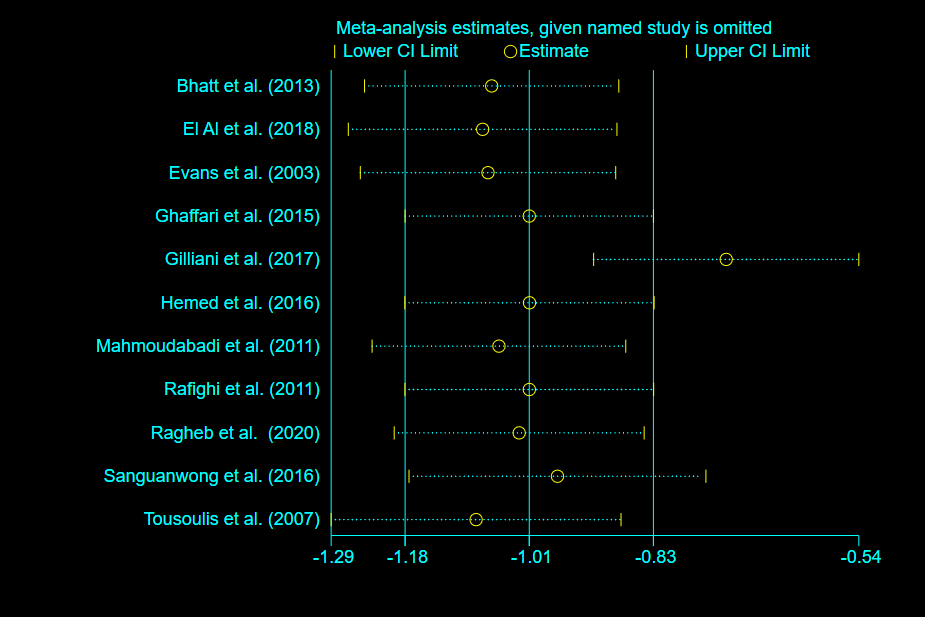


sFigure 6


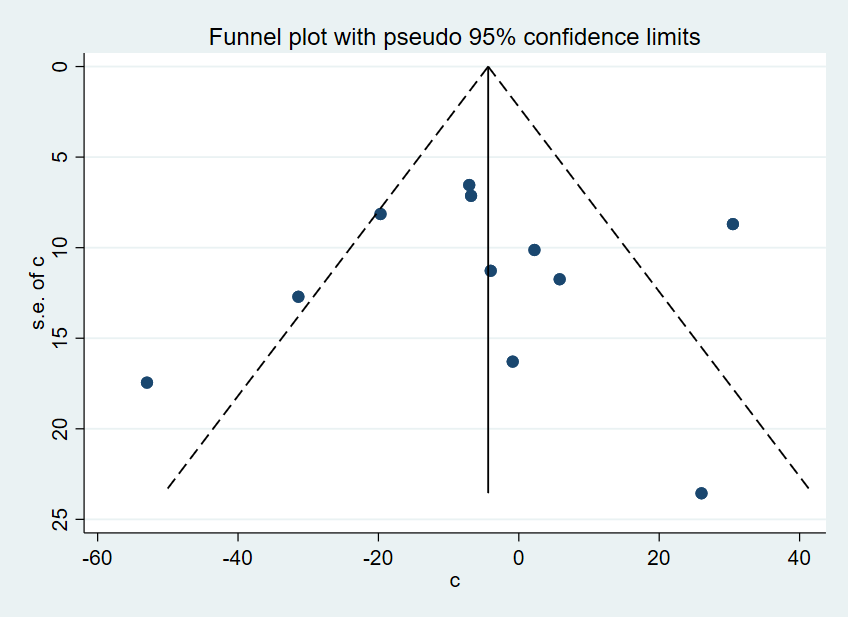


sFigure 7: funnel plot for cholesterol, showing no asymmetry


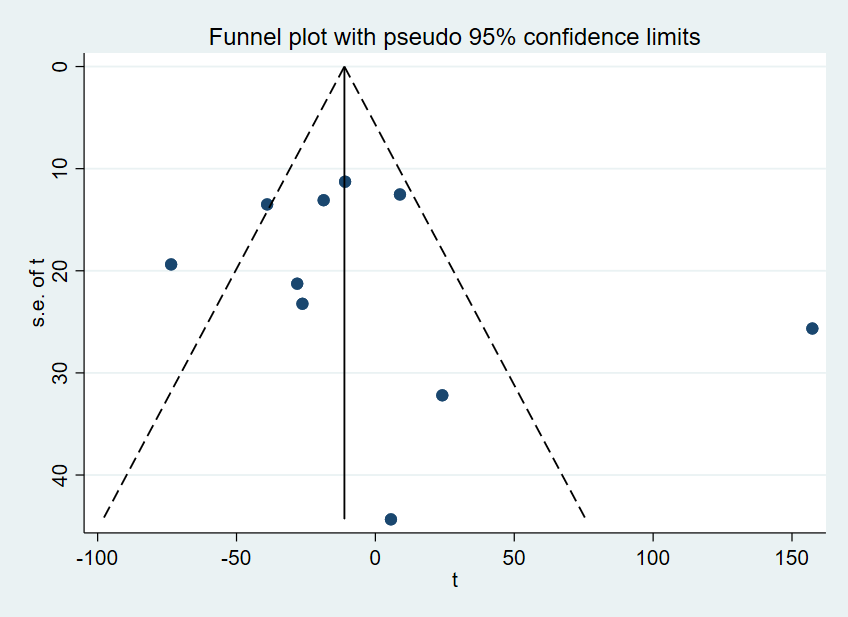


sFigure 8: funnel plot for triglycerides, showing no asymmetry


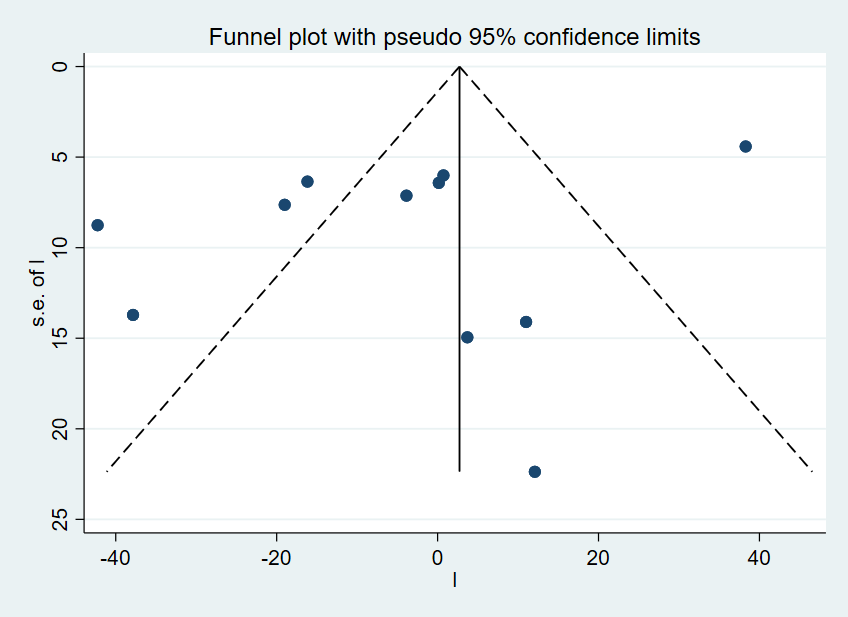


sFigure 9: funnel plot for LDL, showing no asymmetry


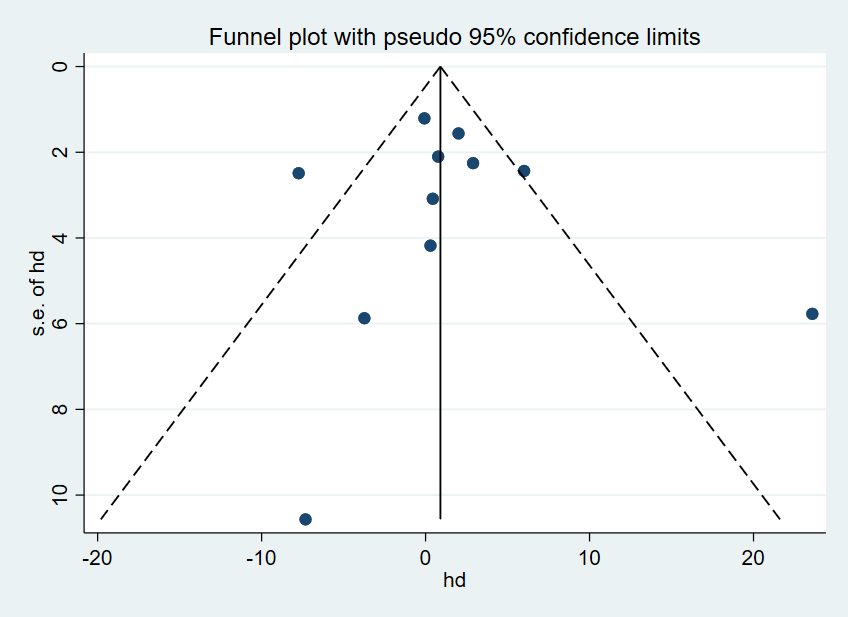


sFigure 10: funnel plot for HDL, showing no asymmetry


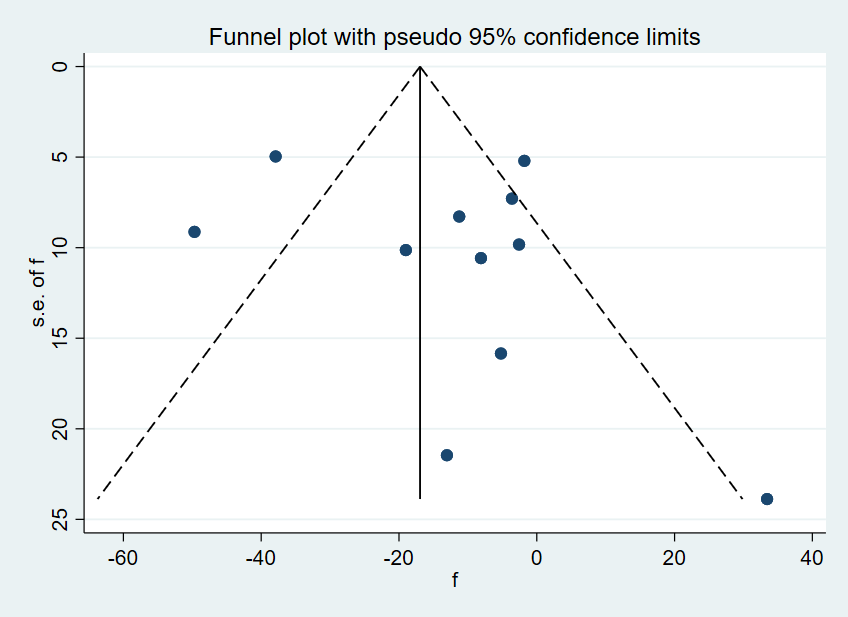


sFigure 11: funnel plot for FBS, showing no visual asymmetry


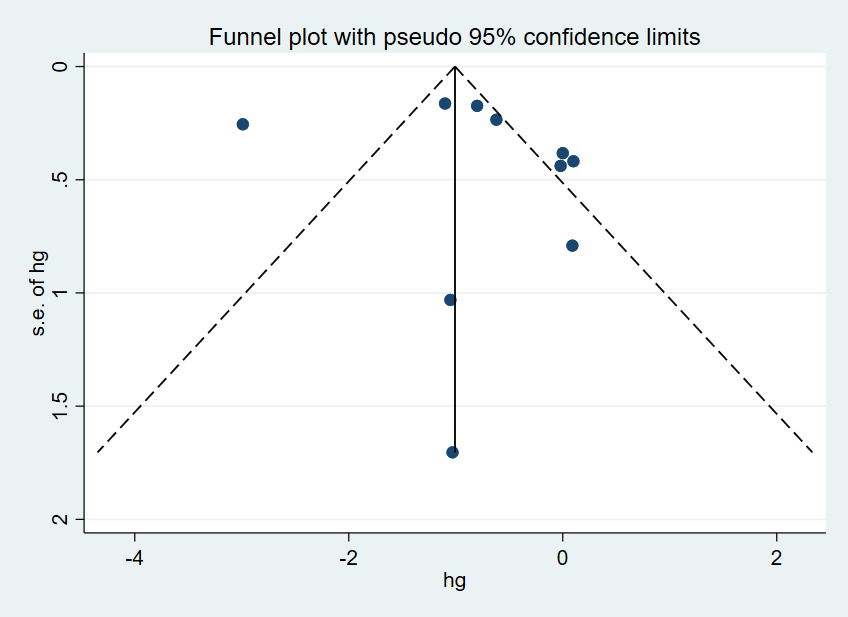


sFigure 12: funnel plot for HgA1C, showing no asymmetry

sTable 7: Publication bias, indicates no significant publication bias for each outcome

|  | Outcome | Egger’s test, p-value |
| --- | --- | --- |
|  | Cholesterol | 0.975 |
|  | triglycerides | 0.353 |
|  | LDL | 0.153 |
|  | HDL | 0.938 |
|  | FBS | 0.074 |
|  | HgA1C | 0.540 |

Table 8: risk of bias for included studies

| Study | Random sequence generation | Allocation concealment | Blinding of participants and personnel | Blinding of outcome assessment | Incomplete outcome data | Other bias |
| --- | --- | --- | --- | --- | --- | --- |
| El Al et al. 2018 | Unclear | Low | Low | High | Low | Low |
| Evans et al. 2003 | Unclear | Unclear | Unclear | Low | low | Low |
| Ghaffari et al. 2015 | Unclear | Low | Unclear | Unclear | Low | Low |
| Gilliani et al. 2017 | Unclear | Low | Low | Unclear | Low | Low |
| Hemed et al. 2016 | High | High | Unclear | Unclear | Low | Low |
| Mahmoudabadi et al. 2011 | Unclear | Low | Low | Low | Low | Low |
| Rafighi et al. 2011 | Low | Low | Unclear | Unclear | Low | Low |
| Ragheb et al. 2020 | Low | Low | Unclear | Unclear | High | Low |
| Sanguanwong et al. 2016 | Low | Low | Low | Low | Low | Low |
| Tousoulis et al. 2007 | Low | Low | High | Low | Low | High |
| Bhatt et al. 2013 | Low | Low | Low | Unclear | Low | Low |
